# Chemical “Butterfly Effect” Explaining the Coordination Chemistry and Antimicrobial Properties of Clavanin Complexes

**DOI:** 10.1021/acs.inorgchem.1c02101

**Published:** 2021-08-12

**Authors:** Adriana Miller, Agnieszka Matera-Witkiewicz, Aleksandra Mikołajczyk, Robert Wieczorek, Magdalena Rowińska-Żyrek

**Affiliations:** † Faculty of Chemistry, University of Wroclaw, F. Joliot-Curie 14, 50-383 Wroclaw, Poland; ‡ Screening Laboratory of Biological Activity Tests and Collection of Biological Material, Faculty of Pharmacy, Wroclaw Medical University, Borowska 211A, 50-556 Wroclaw, Poland

## Abstract

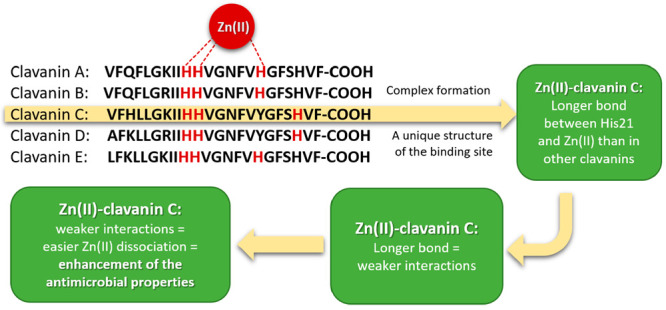

Can a minor difference in the nonmetal binding sequence of antimicrobial clavanins explain the drastic change in the coordination environment and antimicrobial efficiency? This study answers the question with a definite “yes”, showing the details of the bioinorganic chemistry of Zn(II) and Cu(II) complexes with clavanins, histidine-rich, antimicrobial peptides from hemocytes of the tunicate *Styela clava*.

## Abstract 2

Minor differences in clavanin prefolding result in Zn(II) binding site structural rearrangements, leading to impressive enhancement of their antimicrobial properties.

The Zn(II)-clavanin C complex, although its coordination sites are similar to those of other clavanins, has the longest metal–ligand interactions, caused by the presence of the peptide O=C–N–H fragment, which pushes the Zn(II) ion out of its binding pocket. Presumably, this difference is due to a prefolding of the peptide that takes place before Zn(II) binding, and such a structural rearrangement of the metal binding site leads to a remarkable enhancement of the microbiological properties of the Zn(II)-clavanin C complex against a variety of pathogens.

Antimicrobial peptides (AMPs) have recently become a scientifically “hot” topic, appearing as a natural part of the innate immune system to which, with few exceptions, pathogens have developed little resistance compared to traditional antibiotics^1−7^ and often showing synergistic properties to other drugs.^8^ They occur in a variety of organisms, and also the nonmammalian ones show very low toxicity toward mammalian cells.^9,10^

Clavanins, one of the families of AMPs, are 23-amino acid, histidine-rich, cationic peptides^11^ that have a random-coiled conformation in water but show an α-helical structure in membrane-mimicking environments.^12^ There are six types of clavanins (clavanin A–E and clavaspirin), which occur naturally in hemocytes of the tunicate *Styela clava*.^13,14^ At pH 5.5, they inhibit the growth of Gram-positive (*Listeria monocytogenes*, MRSA), Gram-negative bacteria (*Escherichia coli* and *Klebsiella pneumoniae*), and fungi (*Candida albicans*),^11,15^ triggering ongoing studies focused on their use as biofilm-preventing agents^16^ or in bacterial biosensors.^17^

During the over 20-year study on clavanins (mainly clavanin A), various modes of action were proposed.^15,18,19^ Currently, these doubts have been dispelled by Juliano et al.,^20^ who showed three different, pH-dependent mechanisms for clavanin A. The first one is a nonspecific membrane disruption that occurs at neutral pH (7.4).^20^ The second is observed at acidic pH (5.5) when clavanin A binds to DNA and disrupts DNA synthesis, similarly to indolicidin.^20^ The third mechanism, which also occurs at pH 5.5, is assigned not to the “single” clavanin A but to its Zn(II) complex, which cleaves DNA.^20^ Moreover, in the experiment with *E. coli* at pH 5.5, the addition of Zn(II) ions improved clavanin A minimum inhibitory concentration (MIC) from 64 to 4 μM.^15^

It was also emphasized that in the case of clavanin A at pH 5.5, His17 is crucial for both the peptide’s antimicrobial activity and its zinc(II) binding ability; in the α-helical conformation, His17 and His21 are expected to be present on the same side of the helix (*i* and *i* + 4 sites), and this HXXXH motif was suggested to be the primary Zn(II) anchoring site.^15^ Such a motif is also typical for Zn(II)-based nucleases,^21^ and among the six different clavanin sequences (Figure 1), only four (clavanins A, B, and E and clavaspirin) contain the HXXXH pattern.

**Figure 1:**
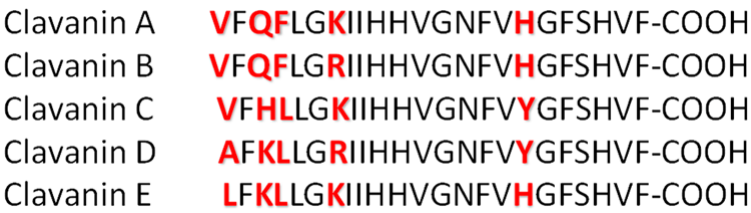
Amino acid sequences of clavanin A–E. APD ID numbers: AP00276–AP00280. The differences between peptides are highlighted. According to Lee et al.^11^ and Lehrer et al.,^14^ tyrosine residues in clavanins C and D could be modified (e.g., methylated).

It is quite well established that, for some AMPs, metal ions act as activity boosters, affecting their charge and/or structure.^22−24^ Interestingly, in the hemocytes of some aquatic invertebrates, quite large amounts of metal ions are found: up to 400 mM Cu(II) and up to 1.2 M Zn(II). This lets us hypothesize that hemocytes of *S. clava* can probably reach similar metal concentrations.^25,26^

On the basis of the clavanin sequential differences, we aimed to establish a coordination pattern, necessary for the biological action of Zn(II)- and Cu(II)-clavanin complexes, and point out the relationship between their metal coordination ability, structure, and antimicrobial mode of action.

Because the available literature data describe C-amidated clavanins at pH 5.5, we decided to focus on the influence of C-terminal deamidation [the free carboxylate group could be an additional Zn(II) binding site; also studies find that the presence of the C-terminal amide group in an AMP can sometimes reduce its antimicrobial properties^27^] on the antimicrobial activity of clavanins and their metal complexes and to perform the studies at physiological pH (7.4), which may be most interesting for possible future applications.

The coordination chemistry of Zn(II) and Cu(II) complexes of clavanins A–E was studied by mass spectrometry (which confirmed the 1:1 stoichiometry of all of the formed complexes; Figures S2A–J and S3A–J), potentiometry, UV–vis, circular dichroism, and NMR spectroscopies and verified by density functional theory (DFT) calculations. Antimicrobial assays showed the effect of the addition of Zn(II) and Cu(II) on the activity of clavanins, and liposome leakage experiments allowed one to suggest whether their mode of action is membrane-disrupting.

The species distributions, as well as p*K*_a_ values and overall stabilities for Zn(II)-clavanin complexes, are very similar (Table S1 and Figure S4A–E); that is why here we discuss only the Zn(II)-clavanin A complex as a representative example.

One, two, and three imidazoles are involved in the binding of Zn(II) in the ZnH_4_L, ZnH_3_L, and ZnH_2_L forms, respectively. The ZnHL species most probably come from deprotonation of the N-terminal amine, which does not take part in the coordination (which was directly confirmed for the Zn(II)-clavanin D complex, where signals from the N-terminal alanine were unaltered in the complex spectra at pH 7.4 with respect to those of the free ligand; Figure S5A). In the ZnL complex, a lysine side chain deprotonates without taking part in the coordination (Figure S4A).

DFT calculations further confirm the 3N-type interactions with imidazole rings for all five Zn(II) complexes at pH 7.4 (Table S2). The complexes of clavanins A, B, and E engage His10, His11, and His17 imidazoles in Zn(II) coordination, while clavanins C and D build complexes using His10, His11, and His21 imidazole rings. This binding mode differs from that previously found for clavanin A at pH 5.5, which engages His17 and His21 in binding.^15^ A change of the coordinating donors with a change of the pH is quite possible and often occurs in the so-called polymorphic binding sites, in which metal ions “move along” the chain of imidazoles involved in binding.^28^

Most interestingly, the Zn(II)-clavanin C complex has the longest metal–ligand bond set, which suggests a rather weak metal–ligand interaction in the series. Such bond elongation can be caused by a unique structure of the binding site; directly below the Zn(II) ion, the O=C–N–H fragment of the peptide backbone aims its H atom almost directly at the metal cation; the H···Zn(II) distance is 2.608 Å, and the N–H··Zn(II) angle is close to linear (160.8°; Figure 2). Such an arrangement of the O=C–N–H fragment close to the positively charged metal results in the longest and weakest metal–ligand bonds and can make the metal dissociation easy in comparison to that in the rest of the complexes in the series. It is noteworth that this kind of interaction, which “pushes” Zn(II) out of its coordination environment, is not observed for the Zn(II)-clavanin D complex, which has the same binding donors as Zn(II)-clavanin C (His10, His11, and His21). The different organization of the binding pocket is most likely due to the prefolding of the clavanin C peptide before the addition of Zn(II) ions. We suggest that these kinds of interactions are also responsible for the (later discussed) impressive microbiological properties of the Zn(II)-clavanin C complex (Figure 3B).

**Figure 2:**
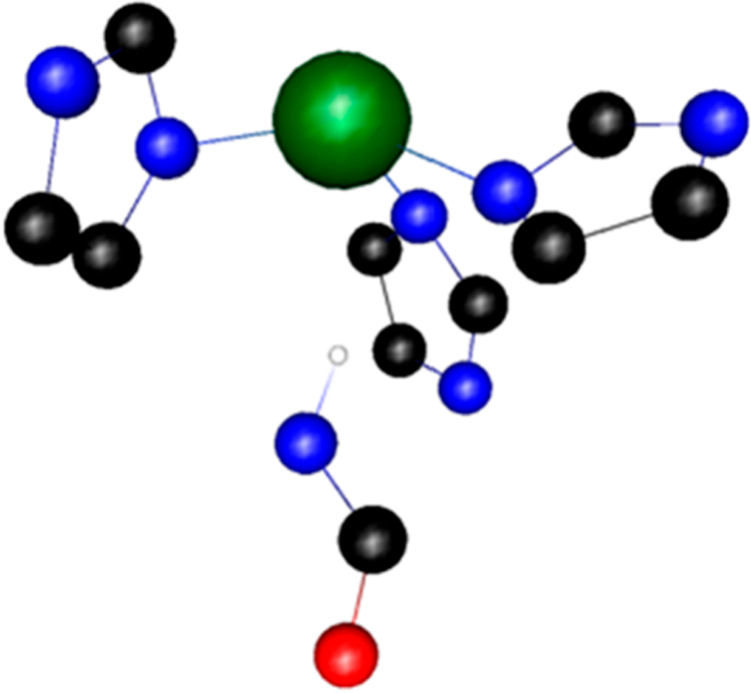
Structure of the binding site of the Zn(II)-clavanin C complex.

**Figure 3:**
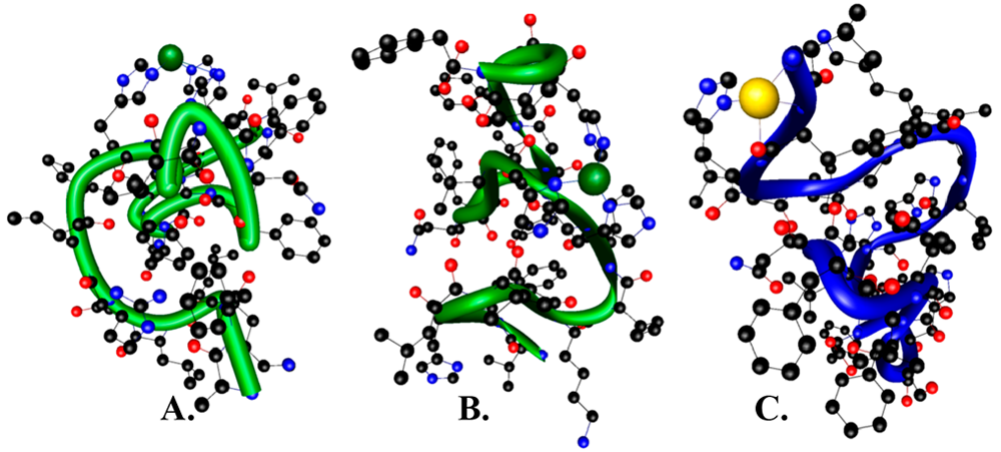
Suggested mode of coordination for (A) Zn(II)-clavanin D, (B) Zn(II)-clavanin C, and (C) Cu(II)-clavanin C complexes.

Detailed descriptions of the pH-dependent distribution forms of Cu(II) complexes are given in Figure S6A–E, supported by Table S1, and spectroscopic data are given in Figures S8A–E and S9A–E. Because the stabilities of Cu(II) complexes with clavanins A, B, D, and E are very similar, we anticipate a similar coordination pattern in all of the mentioned species (Figure S6B,D,E). DFT calculations confirmed the Cu(II) complex coordination modes and additionally showed their precise binding sites. At pH 7.4, clavanins A, B, and E form similar 4N-type connections with Cu(II) via three imidazole rings of His10, His11, and His17, supported by the H17 amide N interaction (Table S3). Clavanin D binds to His10, His11, and His21 and the H11 amide N, and clavanin C (the only clavanin with a histidine in the third position of the peptide sequence) forms a typical albumin-like complex, in which the NH_2_-Xaa-Yaa-His pattern (the ATCUN motif) allows very stable, square-planar complexes to form with Cu(II) and Ni(II);^29^ already in the CuH_2_L form, Cu(II) is bound in an (N_im_, NH_2_, and 2N^–^) coordination mode.

To compare the clavanins’ affinity toward Zn(II) and Cu(II) ions, competition diagrams were prepared (based on the binding constants from Table S1). In the case of Zn(II) complexes, all of them show similar binding affinities (Figure S7A). At pH 5.5, the stabilities of all Cu(II)-clavanin complexes are comparable; the situation changes dramatically at a pH above 6, when Cu(II)-clavanin C (with the previously described so-called albumin-like binding mode) becomes the most stable complex (Figure S7B).

Antimicrobial susceptibility testing was performed on two Gram-negative (*Escherichia coli* ATCC 25922 and *Pseudomonas aeruginosa* ATCC 27853), two Gram-positive (MRSA *Staphylococcus aureus* ATCC 43300 and *Enterococcus faecalis* ATCC 29212), and one fungal strain (*Candida albicans* ATCC 10231). Substantial differences in the MIC values between specific clavanins exist (Table S4). Strikingly, the coordination of Zn(II) strongly enhances the antimicrobial properties of clavanin C against the studied microbes. In the case of other clavanins, most often the presence of metal ions enhances their antimicrobial properties, although this is not a general dependence. The antimicrobial efficiency of Zn(II)-clavanin C is both unexpected and impressive; the MIC obtained against *E. coli* (MIC = 16 μg/mL; Table S4) was lower than the EUCAST breakpoints for amoxicillin–clavulanic acid (penicillins), fosfomycin *p.o.* and *i.v.*, and nitrofurantoin used in standard treatment and equal to those of cefadroxil, cephalexin, and nitroxioline (Table S5). The complex is also active against several clinical *E. coli* strains (Table S6), presents satisfactory results against *E. faecalis*, with MIC equal to that of nitrofurantoin, and is also more potent than fosofmycin *i.v.* and nitrofurantoin against *S. aureus* (MIC = 16 μg/mL; Tables S4 and S5).^30^

A selective metal-enhanced trend is also observed for the activity of both Zn(II) and Cu(II)-clavanin B complexes against *E. faecalis*, with a MIC = 8 μg/mL, which is only twice those of ampicillin and amoxicillin and 8 times less than that of nitrofurantoin. This result is remarkable at least for two reasons: (i) its selectivity toward this pathogen only and (ii) the role of Arg7 in the biological activity of the complex. Although the sequential difference between clavanins A and B is truly minor (a K7R substitution), it leads to changes in the coordination environment (most likely due to prefolding of the peptide before the addition of metal ions). The remarkable antimicrobial effect of the presence of Arg has also previously been described in the literature.^31^ Zn(II)- and Cu(II)-clavanin B complexes were also active against several clinical strains, but no considerably impressive MIC values were obtained (Table S6).

It is noteworthy that no significant cytotoxicity was found against a RPTEC cell line from ECACC collection for any of the studied ligands and their complexes (Table S7).

In conclusion, at physiological pH, clavanins coordinate Zn(II) by three imidazole groups (Figure 3A), always involving His10 and His11 in binding (Table S2). His10 and His11 also participate in the coordination of Cu(II), with the exception of clavanin C, which uses its so-called ATCUN motif (Figure 3C). In the rest of the studied clavanins, at pH 7.4, three imidazoles and one amide group take part in Cu(II) binding.

All Zn(II)-clavanin complexes show similar stabilities, while in the case of the Cu(II) ones, clavanin C is the most potent binding agent. Establishing a direct connection between the bioinorganic chemistry of the metal-clavanin complexes and their antimicrobial activity is far from trivial, and it certainly does not depend on the thermodynamic stability of the complexes (and therefore is not based on nutritional immunity). Liposome experiments confirmed that both the free peptides and their complexes show average membrane disrupting ability, further suggesting their mode of action to be intracellular (Figure S10A–F).

Good or moderate antifungal activity of the whole clavanin family is observed, which is often metal-enhanced. The most spectacular metal enhancement of the antimicrobial properties is seen for the Zn(II)-clavanin C complex, which is quite surprising. On the basis of the literature data,^32,33^ we would have expected such an effect for the ATCUN-bound Cu(II) but not Zn(II) complexes. DFT calculations came with a possible explanation of this phenomenon: in the case of the Zn(II)-clavanin C complex, a unique structure of the binding site is observed in which the O=C–N–H fragment of the peptide backbone is present directly below the Zn(II) ion, with the H atom “pushing” the Zn(II) ion out of its binding pocket, resulting in a long metal–ligand bond that can make Zn(II) dissociation from the complex easier than that of the rest of the complexes. The different organization of the binding pocket is most likely due to the prefolding of clavanin C, which takes place before addition of the Zn(II) ions, acting as a “butterfly effect” for the later Zn(II) complex structure and its surprisingly enhanced antimicrobial properties.

## Supporting Information Available

Experimental section and addditional figures and tables (PDF)
