# Supplementary material for: Chemical “Butterfly Effect” Explaining the Coordination Chemistry and Antimicrobial Properties of Clavanin Complexes
Source: Inorg Chem. 2021 Aug 12;60(17):12730–4. doi: 10.1021/acs.inorgchem.1c02101 (PMC8424629; doi:10.1021/acs.inorgchem.1c02101)
Supplement: Supplementary file 1 — ic1c02101_si_001.pdf [file ic1c02101_si_001.pdf]

Electronic Supplementary Information for:

# Chemical "Butterfly Effect" Explaining the Coordination Chemistry and Antimicrobial Properties of Clavanin Complexes

Adriana Miller[a], Agnieszka Matera-Witkiewicz[b*], Aleksandra Mikołajczyk[b], Robert Wieczorek[a], Magdalena Rowińska – Żyrek[a*]

a) Faculty of Chemistry, University of Wroclaw, F. Joliot – Curie 14, 50-383 Wroclaw, Poland

b) Screening Laboratory of Biological Activity Tests and Collection of Biological Material, Faculty of Pharmacy, Wroclaw Medical University, Borowska 211A, 50-556 Wroclaw, Poland

# Table of Contents

1. Experimental
   - Materials (page 5)
   - Mass spectrometric measurements (page 5)
   - Potentiometric measurements (page 5)
   - Spectroscopic measurements (page 5)
   - Liposome preparation and leakage assay (page 6)
   - DFT calculations (page 6)
   - Antimicrobial activity assay of peptide and peptide-metal ion complex system (page 7)
   - Neutral Red cytotoxicity assay (page 8)
2. Results
   - Mass spectrometry (page 8)
   - Protonation of the ligands (page 16)
   - Zn(II) complexes (page 17)
   - Cu(II) complexes (page 20)
   - *In vitro* cytotoxicity studies (page 30)
   - Membrane disrupting ability (page 31)
3. Figures
   - Fig. S1 The clavanin A, B, C, D and E (from left to right) MD folded structures, blue tubes follow backbones (page 7)
   - Fig. S2. ESI-MS spectra of: A) Zn(II)-Clavanin A; B) Cu(II)-clavanin A; C) Zn(II)-clavanin B; D) Cu(II)-clavanin B; E) Zn(II)-clavanin C; F) Cu(II)-clavanin C; G) Zn(II)-clavanin D; H) Cu(II)-clavanin D; I) Zn(II)-clavanin E; J) Cu(II)-clavanin E; $M^{2+}$:L molar ratio = 1:1, pH = 7.4 (pages 9-12)
   - Fig. S3. Isotopic distribution of A) Zn(II)-clavanin A; B) Cu(II)-clavanin A; C) Zn(II)-clavanin B; D) Cu(II)-clavanin B; E) Zn(II)-clavanin C; F) Cu(II)-clavanin C; G) Zn(II)-clavanin D; H) Cu(II)-clavanin D; I) Zn(II)-clavanin E; J) Cu(II)-clavanin E; $M^{2+}$:L molar ratio = 1:1, pH = 7.4 (pages 13-16)
   - Fig. S4. Distribution diagrams for the formation of: A) Zn(II) complex with clavanin A; B) Zn(II) complex with clavanin B; C) Zn(II) complex with clavanin C; D) Zn(II) complex with clavanin D; E) Zn(II) complex with clavanin E; T=298K, I= 40 mM SDS, $[M^{2+}]=0.5.10^{-3}$ M; $M^{2+}$:L molar ratio = 1:1 (pages 18-19)
   - Fig. S5. 1H–1H TOCSY NMR spectra of a fragment of the clavanin D (gray) and the complex (red) with Zn(II) (A) or Cu(II) (B); [clavanin D] = 3 mM; Zn(II):L molar ratio 1:1, Cu(II):L molar ratio 1:0.2; pH = 3, T = 298 K, I = 40 mM dSDS in 90 % $H_2O$ and 10 % $D_2O$ solution (pages 19-20)
   - Fig. S6. Distribution diagrams for the formation of: A) Cu(II) complex with clavanin A; B) Cu(II) complex with clavanin B; C) Cu(II) complex with clavanin C; D) Cu(II) complex with clavanin D; E) Cu(II) complex with clavanin E; T=298K, I= 40 mM SDS, $[M^{2+}]=0.5.10^{-3}$ M; $M^{2+}$:L molar ratio = 1:1 (pages 22-23)

- Fig. S7. Competition plot between clavanin A, B, C, D, E and Zn(II) (A) or Cu(II) (B), describing complex formation at different pH values in a hypothetical situation, in which equimolar amounts of the six reagents are mixed (page 24)
- Fig. S8. CD spectra of Cu(II) complexes with: A) clavanin A; B) clavanin B; C) clavanin C; D) clavanin D; E) clavanin E; in pH range 2-11. Conditions: T = 298 K, I = 40 mM SDS, [Cu(II)] = [clavanin A] = [clavanin B] = [clavanin C] = [clavanin D] = [clavanin E] = 0.001 M (pages 25-26)
- Fig. S9. UV-Vis spectra of Cu(II) complexes with: A) clavanin A; B) clavanin B; C) clavanin C; D) clavanin D; E) clavanin E; in pH range 2-11. Conditions: T = 298 K, I = 40 mM SDS, [Cu(II)] = [clavanin A] = [clavanin B] = [clavanin C] = [clavanin D] = [clavanin E] = 0.001 M (pages 26-28)
- Fig. S10. Percentage of disrupted liposomes by: A) clavanin A; B) clavanin B; C) clavanin C; D) clavanin D; E) clavanin E. Diagram F) presents comparison of all tested clavanins. All samples were prepared in HEPES buffer (10 mM HEPES, 150 mM NaCl, pH = 7.4). Conditions: T = 298 K, [Cu(II)] = [Zn(II)] = [clavanin A] = [clavanin B] = [clavanin C] = [clavanin D] = [clavanin E] = 5 µM (pages 31-33)

4. Tables
   - Table S1. Potentiometric data for proton, Zn(II) and Cu(II) complexes with VFQFLGKIIHHVGNFVHGFSHVF-COOH, VFQFLGRIIHHVGNFVHGFSHVF-COOH, VFHLLGKIIHHVGNFVYGFSHVF-COOH, and AFKLLGRIIHHVGNFVYGFSHVF-COOH and LFKLLGKIIHHVGNFVHGFSHVF-COOH (page 17)
   - Table S2. Metal – ligand distances in angstroms for clavanin A, B, C, D, E and Zn(II) complexes (page 20)
   - Table S3. Metal – ligand distances in angstroms for clavanin A, B, C, D, E and Cu(II) complexes (page 23)
   - Table S4. In vitro antibacterial activity of clavanins A, B, C, D and E determined as a minimal inhibitory concentration (MIC) (µg/mL); n/d, not determined. Experiments were performed for all compounds and their metal complexes according to the ISO 20776-1:2019 and ISO 16256:2012. No MIC value was determined for *Pseudomonas aeruginosa* ATCC 27853. No MBC/MFC activity was observed after performing modified Richard's method. Bolded values represent concentrations lower than or equal to established by EUCAST breakpoints for selected antimicrobial agents characteristic for given bacterial families (page 28)
   - Table S5. Examples of MIC breakpoints values from EUCAST/2021/01/01 for bacteria (page 29)
   - Table S6. In vitro antibacterial activity of clavanins against clinical strains of indicated species determined as a minimal inhibitory concentration (MIC) (µg/mL); n/d, not determined. No antimicrobial activity was determined for tested clinical strains of *Staphylococcus aureus* (page 29)
   - Table S7. Cell viability using Neutral red uptake assay (NR) after 24 and 48 h incubation with unmodified and C-terminal amidated clavanins and clavanin-

metal ion systems. Used compounds and their concentration were selected after antimicrobial assay (page 30)

– Table S8. Antibiotic resistance in indicated species of Gram-negative bacteria (page 34)
– Table S9. Antibiotic resistance in indicated species of Gram-positive bacteria (page 34)

# Experimental

## Materials

All peptides (VFQFLGKIIHHVGNFVHGFSHVF-COOH, VFQFLGRIIHHVGNFVHGFSHVF-COOH, VFHLLGKIIHHVGNFVYGFSHVF-COOH, AFKLLGRIIHHVGNFVYGFSHVF-COOH, LFKLLGKIIHHVGNFVHGFSHVF-COOH) were purchased from KareBay Biochem (USA) (certified purity: 98 %) and were used as received.

The carbonate-free stock solutions of 0.1 M NaOH were purchased from Sigma-Aldrich and then potentiometrically standardized with the primary standard potassium hydrogen phthalate (99.9 % purity).

## Mass spectrometry

High-resolution mass spectra were obtained on a Bruker Apex Ultra FT-ICR (Bruker Daltonik, Bremen, Germany), equipped with an Apollo II electrospray ionization source with an ion funnel. The mass spectrometer was operated in the positive ion mode. The instrumental parameters were as follows: scan range m/z 100–2000, dry gas – nitrogen, temperature 473 K, and ion energy 5 eV. The capillary voltage was optimized to the highest S/N ratio and it was 4200 V. The samples were prepared in a 1 : 1 methanol–water mixture with a $M^{2+}$ : L molar ratio 1 : 1, [ligand] = 3 x $10^{-4}$ M, pH 7.4. The samples were infused at a flow rate of 3 µL $min^{-1}$. The instrument was calibrated externally with a Tunemix™ mixture (Bruker Daltonik, Germany) in quadratic regression mode. Data were processed using the Bruker Compass DataAnalysis 4.0 program. The mass accuracy for the calibration was better than 5 ppm, enabling together with the true isotopic pattern (using SigmaFit) an unambiguous confirmation of the elemental composition of the obtained complex.

## Potentiometry

Stability constants for proton, Zn(II) and Cu(II) complexes were calculated from pH-metric titration curves carried out over the pH range 2-11 at T = 298 K in water solution of 4 mM $HClO_4$ and ionic strength 40 mM (SDS), using a total volume of 3 ml. The potentiometric titrations were performed using a Metrohm Titrando 905 titrator and a Mettler Toledo InLab Micro combined pH electrode. The thermostabilized glass-cell was equipped with a magnetic stirring system, a microburette delivery tube and an inlet–outlet tube for argon. Solutions were titrated with 0.1 M carbonate-free NaOH. The electrodes were calibrated daily for hydrogen ion concentration by titrating $HClO_4$ with NaOH under the same experimental conditions as above. The purities and the exact concentrations of the ligand solutions were determined by the Gran method.[1] The ligand concentration was 0.5 mM. The Zn(II) and Cu(II) to ligand ratio was 1 : 1.

The standard potential and the slope of the electrode couple were computed by means of Glee[2] program. The HYPERQUAD 2006[3] program was used for the stability constant calculations. The standard deviations were computed by HYPERQUAD 2006 and refer to random errors only. The constants for the hydrolytic Zn(II) species were used in these calculations. The speciation and competition diagrams were computed with the HYSS program.

## Spectroscopic measurements

The absorption spectra were recorded on Varian Cary300 Bio spectrophotometer, in the range 200–800 nm, using a quartz cuvette with an optical path of 1 cm. Circular dichroism (CD) spectra were registered on a Jasco J-1500 CD spectrometer in the 200–800 nm range, using a quartz cuvette with an optical path of 1 cm or with a

cuvette with an optical path of 0.01 cm in the wavelength range 180–300 nm. The solutions were prepared in a water solution of 4 mM $HClO_4$ at ionic strength I = 40 mM (SDS). The concentrations of solutions used for spectroscopic studies were similar to those in the potentiometric experiments; Cu(II) : ligand ratio was also 1:1. The UV-Vis and CD spectroscopic parameters were calculated from the spectra obtained at the pH values corresponding to the maximum concentration of each particular species, based on distribution diagrams.

NMR spectra were recorded at 14.1 T on a BrukerAvance III 600 MHz equipped with a Silicon Graphics workstation. The temperatures were controlled with an accuracy of ±0.1 K. Suppression of the residual water signal was achieved by excitation sculpting, using a selective square pulse on water 2 ms long. All the samples were prepared in 40 mM dSDS dissolved in a 90 % $H_2O$ and 10 % $D_2O$ solution. The proton resonance assignment was accomplished by 2D $^1H$–$^1H$ total correlation spectroscopy (TOCSY), carried out with standard pulse sequences. Spectral processing and analysis was performed using a Bruker TOPSPIN 2.1 and Sparky. Samples of the analyzed complexes were prepared by adding metal ions to an acidic solution of a 3 mM ligand (pH 3), and the pH was then increased to the desired higher value.

## Liposome preparation and leakage assay

Fluorescence spectra were recorded in a kinetic mode on a Varian Cary Eclipse spectrofluorometer using a quartz fluorescence cuvettes with an optical path of 1 cm.

10 mg of lipids (DOPC:DOPA:Cholesterol 30:30:40 % mol %) were dissolved in 1 ml of chloroform and then dried under a stream of air for 4 h. The dry lipid film was hydrated with a solution of 6-carboxyfluorescein in HEPES buffer (20 mM 6-carboxyfluorescein, 10 mM HEPES, 150 mM NaCl, pH = 7.4), sonicated and subjected to three freeze-thaw cycles (-15 $^oC$ – 60 $^oC$).

To obtain homogenous liposomes, the liposomal suspension was extruded for 70 times through the polycarbonate membranes with a pore size 100 nm, mounted on Avanti Mini-Extruder (Avanti Polar Lipids). To remove untrapped 6-carboxyfluorescein, the liposomal suspension was filtered twice through a Sephadex-G50 column.

5 μM solutions of peptides in HEPES buffer were prepared. Each sample contained 1980 μl of peptide solution and 20 μl of liposomes. To obtain 100 % of dye leakage, after each measurement 60 μl of 1 % Triton X100 was added to the sample. 1980 μl of HEPES buffer and 20 μl of liposomes were measured to check the passive dye leakage. Each experiment was carried out for 15 minutes with excitation and emission wavelength of 492 nm and 512 nm, respectively.

To assess the percent of disrupted liposomes, results were analyzed with the following formula:

$$Disruption_{time} = [(F_{15} - F_0)/(F_{100\%} - F_0)] \times 100\%$$

where $F_{15}$ – the intensity of fluorescence after 15 minutes, $F_0$ – the intensity of fluorescence in t = 0 s, $F_{100\,\%}$ - the intensity of fluorescence after Triton X100 addition.[4,5]

## DFT calculations

Computational methods of theoretical chemistry have been used as useful tool to predict structure and stability of the ligands and complexes.[6–11] Molecular orbital studies on 1:1 complexes of Cu(II) and Zn(II) ions with clavanin A, B, C, D and E have been done on the DFT level of theory with IEFPCM[12] solvent (water) model, introduced upon potential energy surface investigation. The starting structure of the peptide for DFT

calculations was generated on the basis of the amino acid sequence after 75 ps simulation at 300 K, without cutoffs using BIO+ implementation of CHARMM force field as shown in Fig. S0. DFT calculations were performed with Gaussian 09 C.01[13] suite of programs using the ωB97X-D[14] long-range corrected hybrid density functional with damped atom-atom dispersion corrections was used with a double-ζ 6-31G(d,p) basis set containing polarization functions. The metal-ligand distances in the starting geometries were arbitraly chosen with $R_{N..Cu}$=1.87A and $R_{N..Zn}$=1.95A respectively. All presented structures were fully optimized and all presented complexes are thermodynamically stable.

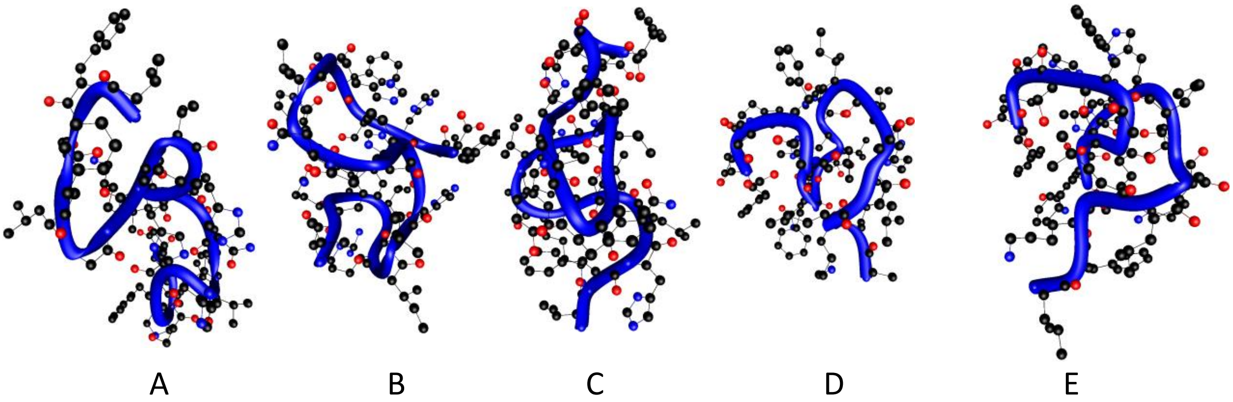


**Fig. S1.** The clavanin A, B, C, D and E (from left to right) MD folded structures, blue tubes follow backbones.

## Antimicrobial activity assay of peptide and peptide-metal ion complex system

Five reference strains from ATCC collection (*Pseudomonas aeruginosa* 27853*, Escherichia coli 25922, Staphylococcus aureus 43300, Enterococcus faecalis 29212 and Candida albicans 10231*) and clinical strains isolated from specific parts of the human body (*Escherichia coli, Enterococcus faecalis, Staphylococcus aureus MRSA*; see Table S8, S9 where antibiotic resistance is presented) were used for antimicrobial activity assay. The antimicrobial effect of analysed peptides/complexes was performed according to the standard protocol using microdilution method with spectrophotometric measurement (λ = 580 nm at starting point and after 24 h)[15] according to the ISO standard 20776-1:2019,[16] ISO standard 16256:2012[17] and modified Richard's method.[18–20]

Stock peptide solutions were prepared in 0.9 % NaCl four times concentrated. Serial dilutions of ligand/complex solution were made on 96-well microplates in the range between 0.5 µg/mL and 256 µg/mL. Tryptone Soya Agar (TSA) plates were inoculated with microbial strains from glycerol stocks. After 24h/37 $^{o}$C incubation (for bacteria) or 24h/25 $^{o}$C (for fungus), a proper density of bacterial and fungal suspension was prepared using a densitometer (final inoculum (5x10$^{5}$ CFU/mL) was prepared in Tryptic Soy Broth (TSB)). A positive (TSB with 0.23 % NaCl + strain) and negative control (TSB with 0.23 % NaCl) were also included in the test. Spectrophotometric solubility control of each peptide and peptide-metal ion system was also performed. For each strain, the validation process was performed using following antibacterial/antifungal agents: levofloxacin, gentamicin, amphotericin B, according to the EUCAST examination. Minimal Inhibitory Concentration (MIC) was determined as the lowest concentration of an antimicrobial agent that decreased the measured microbial growth to 50 % as referred to positive control. Obtained MIC values were for *Pseudomonas aeruginosa* 27853: levofloxacin 1 µg/mL, *Escherichia coli* 25922: gentamicin 2 µg/mL, *Staphylococcus aureus* 43300: levofloxacin 1 µg/mL, *Enterococcus faecalis* 29212: levofloxacin 4 µg/mL, *Candida albicans* 10231: amphotericin B 1 µg/mL.

Microplates were incubated at 37±1 °C or 25±1 °C for 24 hours on the shaker (500 rpm). After this, the spectrophotometric measurement was performed at 580 nm and then 50 µL aliquots of 1 % (m/v) 2,3,5-triphenyltetrazolium chloride (TTC) solution were added into each well. TTC is a chemical indicator which is converted into red formazan crystals in living microbial cells. MBC/MFC can be observed as the lowest concentration required to kill a particular microbial strain, determined by visual analysis after 24h incubation with TTC (did not change the colour to pink). Thanks to both methods, MIC and MBC or MFC can be determined. The results are presented in Tables S4, S6.

### Neutral Red cytotoxicity assay

For each peptide and peptide-metal ion system, where the antimicrobial activity was determined, a Neutral Red (NR) cytotoxicity assay was performed using human primary renal proximal tubule epithelial cells (RPTEC) from ECACC collection. The experiment was performed according to ISO:10993 guidelines (Biological evaluation of medical devices; Part 5: Tests for in vitro cytotoxicity; Part 12: Biological evaluation of medical devices, sample preparation and reference materials (ISO 10993-5:2009 and ISO/IEC 17025:2005). A standard protocol for the NR assay was used from *Nature Protocol.*[21] MEMα supplemented with 10 % FBS, 2 mM L-glutamine and suitable amount of antibiotics (amphotericin B, gentamycin) was used for the experiment. Investigated concentrations used in experiment are presented in Table S7. Also Cu(II) and Zn(II) salt solutions were checked to eliminate potential cytotoxic effect of metal ions. Stock peptide solutions were prepared in 0.9 % NaCl and then 100 times diluted in the medium. After adding proper mixtures of testing compounds and cells ($1x10^4$) into each well, plates were incubated for 48 and 72 h in 5 % $CO_2$ at 37 $^oC$. Next, medium was removed and 100 µL of NR solution (40 µg/mL) was added to each well followed by incubation for 2 h at 37 $^oC$. After removing the dye, wells were rinsed with PBS and left to dry. Then, NR destain solution (1 % glacial acetic acid, 50 % of 96 % ethanol and 49 % of deionized water; v/v) was added to each well. The plates were shaken (30 min, 500 rpm) until NR was extracted from the cells and formed a homogenous solution. The absorbance was measured using microplate reader at 540 nm. As a negative control untreated cells were considered as 100 % of potential cellular growth. Furthermore, cells incubated with 1 µM staurosporine were used as a positive control. The experiment results are presented in Table S7.

## Results

### Mass spectrometry

In the Zn(II)- clavanin A (Zn(II)- VFQFLGKIIHHVGNFVHGFSHVF-COOH) mass spectra (Fig. S2A), three the most intensive peaks can be assigned to the free ligand ($m/z$ = 889.8; $z$ = 3+), its potassium adduct ($m/z$ = 904.1; $z$ = 3+), and the potassium adduct of the zinc(II) complex ($m/z$ = 923.5; $z$ = 3+). In the spectra of Cu(II) complex of the clavanin A (Fig. S2B), signals from the free ligand ($m/z$ = 667.6; $z$ = 4+) and its complex ($m/z$ = 683,1; $z$ = 4+) are observed.

In the Zn(II)-clavanin B (Zn(II)- VFQFLGRIIHHVGNFVHGFSHVF-COOH) mass spectra (Fig. S2C), the prevailing signals which come from the free ligand ($m/z$ = 674.6; $z$ = 4+), the sodium adduct of the ligand ($m/z$ = 680.1; $z$ = 4+) and Zn(II) complex ($m/z$ = 690.3; $z$ = 4+) are visible. In the case of Cu(II) complex of clavanin B (Fig. S2D), beside the signal which corresponds to the free ligand ($m/z$ = 674.6; $z$ = 4+), its sodium adduct ($m/z$ = 680.1; $z$ = 4+), the Cu(II) complex ($m/z$ = 690.1; $z$ = 4+) are observed.

In the mass spectra of the Zn(II)-clavanin C (Zn(II)- VFHLLGKIIHHVGNFVYGFSHVF-COOH) (Fig. S2E), peaks with the highest intensity can be assigned to the free ligand ($m/z$ = 667.9; $z$ = 4+), its sodium adduct ($m/z$ = 673.4; $z$= 4+) and Zn(II) complex ($m/z$ = 683.3; $z$ = 4+). In the spectra of the same ligand with Cu(II) (Fig. S2F), the

observed signals come from the free ligand (*m/z* = 667.9; *z* = 4+), the sodium adduct of the ligand (*m/z* = 673.4; *z* = 4+) and Cu(II) complex (*m/z* = 683.3; *z* = 4+).

In the Zn(II)-clavanin D (Zn(II)- AFKLLGRIIHHVGNFVYGFSHVF-COOH) mass spectra (Fig. S2G), three intensive signals are visible. First corresponds to the free ligand (*m/z* = 665.6; *z* = 4+), second – to its sodium adduct (*m/z* = 671.1; *z* = 4+) and third to the Zn(II) complex (*m/z* = 681.3; *z* = 4+). In the spectra of the Cu(II)-clavanin D (Fig. S2H), the observed peaks can be assigned to the free ligand (*m/z* = 665.6; *z* = 4+), its sodium adduct (*m/z* = 671.1; *z* = 4+), and the Cu(II) complex (*m/z* = 681.1; *z* = 4+).

In the Zn(II)-clavanin E (Zn(II)- LFKLLGKIIHHVGNFVHGFSHVF-COOH) mass spectra (Fig. S2I), signals from the free ligand (*m/z* = 662.6; *z* = 4+), the sodium adduct of the ligand (*m/z* = 668.1; *z* = 4+) and Zn(II) complex (*m/z* = 678.1; *z* = 4+) are observed. In the spectra of the same ligand with Cu(II) (Fig. S2J), peaks corresponds to the free ligand (*m/z* = 662.6; *z* = 4+), its sodium adduct (*m/z* = 668.1; *z* = 4+) and the Cu(II) complex (*m/z* = 678.4; *z* = 4+).

All simulated mass spectra for the Zn(II) and Cu(II) complexes are in perfect agreement with experimental ones (Fig. S3 A-J).

A.

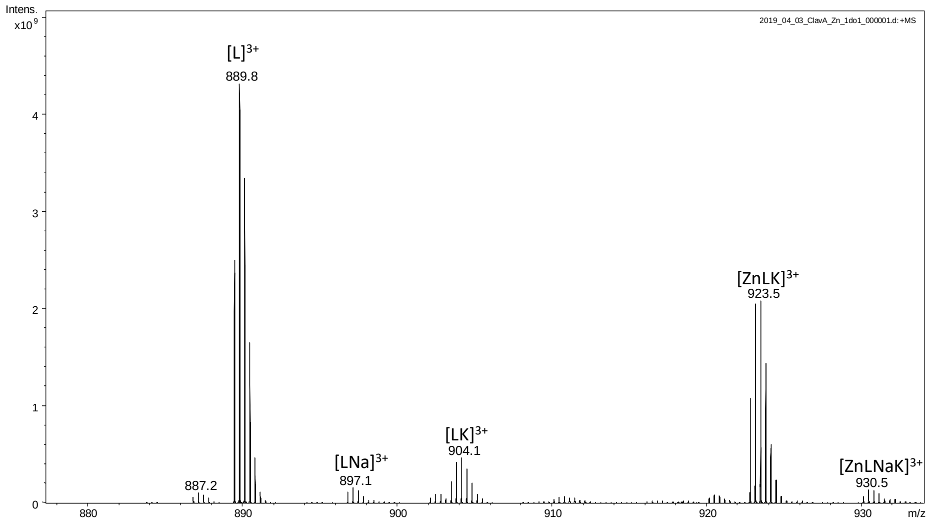


B.

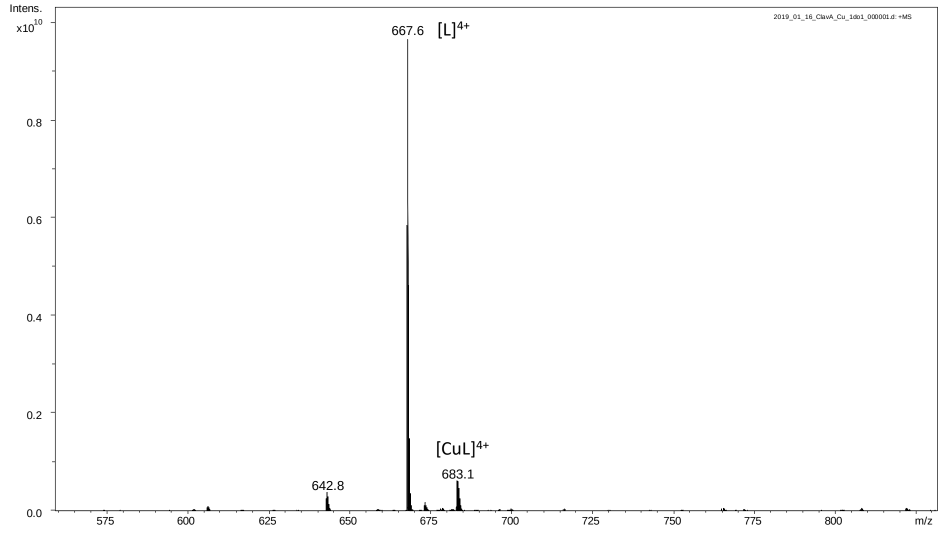

C.

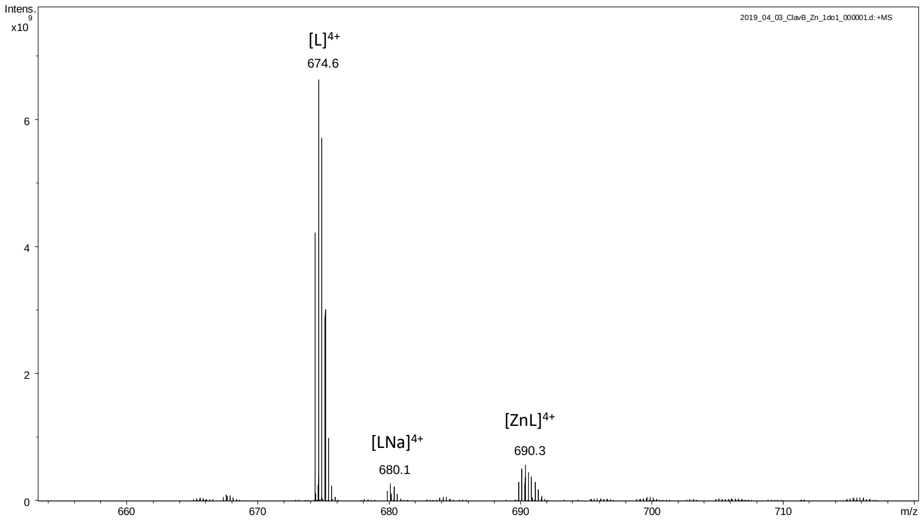
Intens.
x10^9
2019_04_03_ClavB_Zn_1do1_000001.d: +MS
[L]4+
674.6
[LNa]4+
680.1
[ZnL]4+
690.3
0
2
4
6
660
670
680
690
700
710
m/z

D.

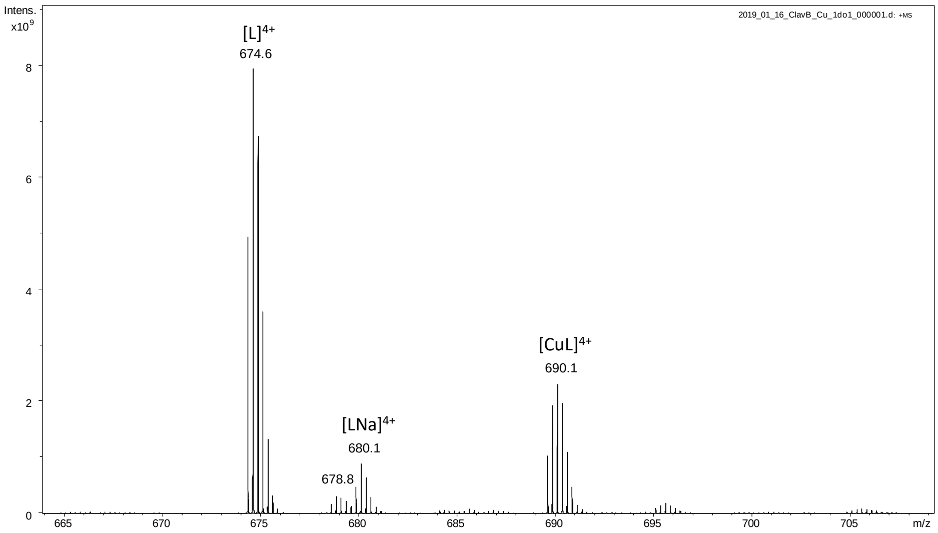
Intens.
x10^9
2019_01_16_ClavB_Cu_1do1_000001.d: +MS
[L]4+
674.6
[LNa]4+
680.1
678.8
[CuL]4+
690.1
0
2
4
6
8
665
670
675
680
685
690
695
700
705
m/z

E.

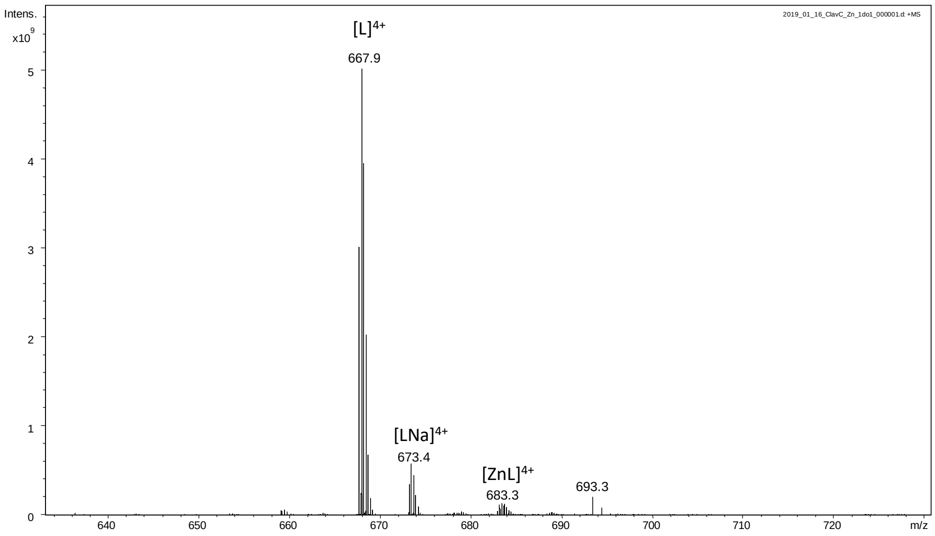
Intens.
x10^9
2019_01_16_ClavC_Zn_1do1_000001.d: +MS
[L]4+
667.9
[LNa]4+
673.4
[ZnL]4+
683.3
693.3
0
1
2
3
4
5
640
650
660
670
680
690
700
710
720
m/z

F.

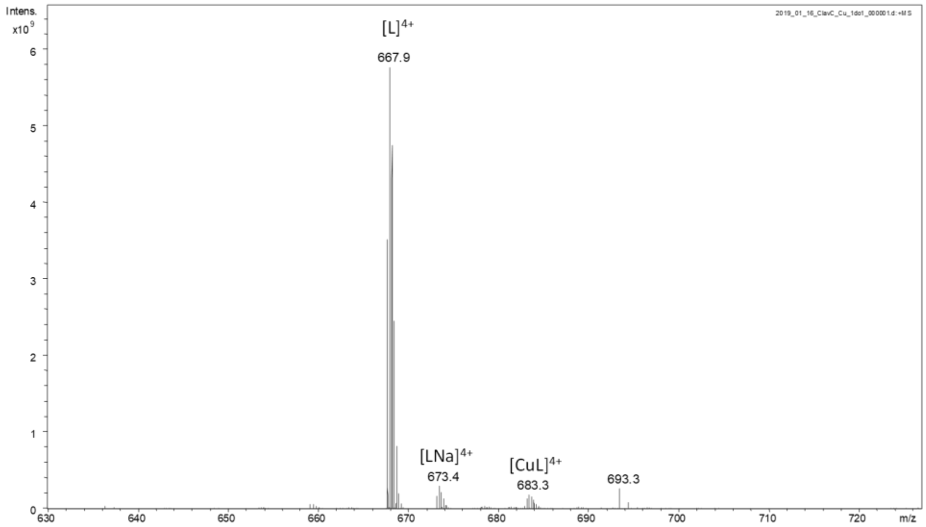
Intens.
x10^9
[L]4+
667.9
[LNa]4+
673.4
[CuL]4+
683.3
693.3
m/z


G.

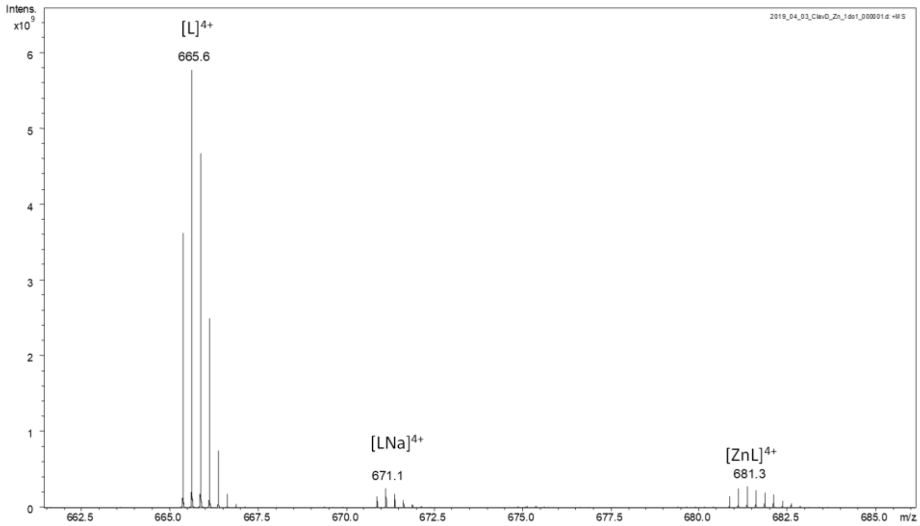
Intens.
x10^9
[L]4+
665.6
[LNa]4+
671.1
[ZnL]4+
681.3
m/z


H.

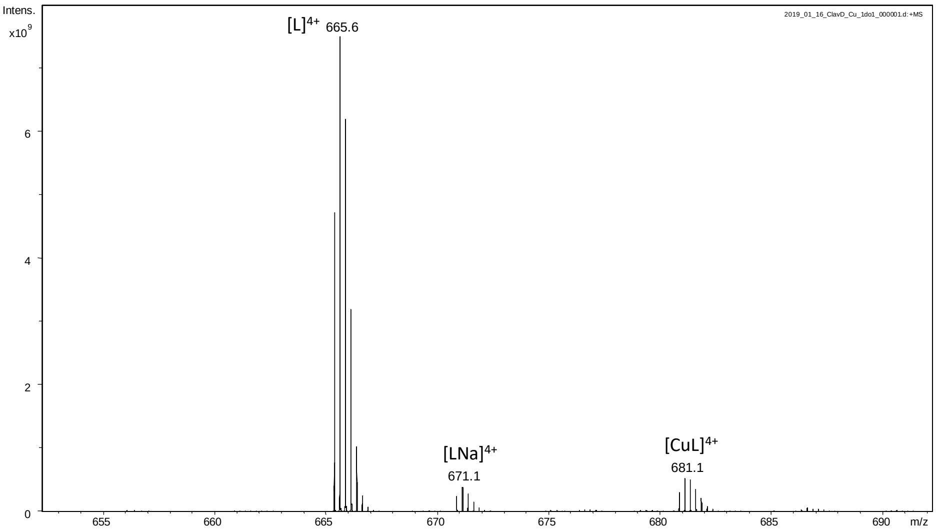
Intens.
x10^9
[L]4+ 665.6
[LNa]4+
671.1
[CuL]4+
681.1
m/z

I.

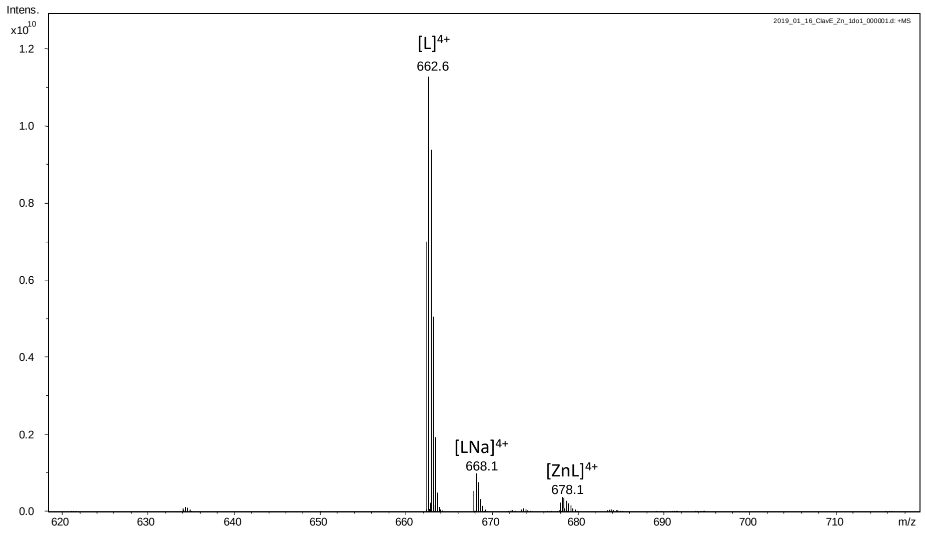


J.

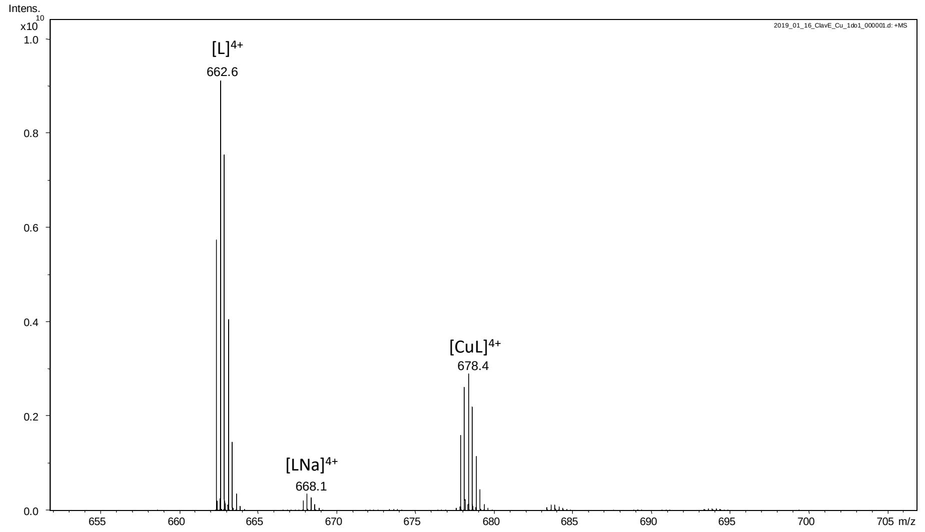


**Fig. S2.** ESI-MS spectra of: A) Zn(II)-clavanin A; B) Cu(II)-clavanin A; C) Zn(II)-clavanin B; D) Cu(II)-clavanin B; E) Zn(II)-clavanin C; F) Cu(II)-clavanin C; G) Zn(II)-clavanin D; H) Cu(II)-clavanin D; I) Zn(II)-clavanin E; J) Cu(II)-clavanin E; $M^{2+}$:L molar ratio = 1:1, pH = 7.4

A.

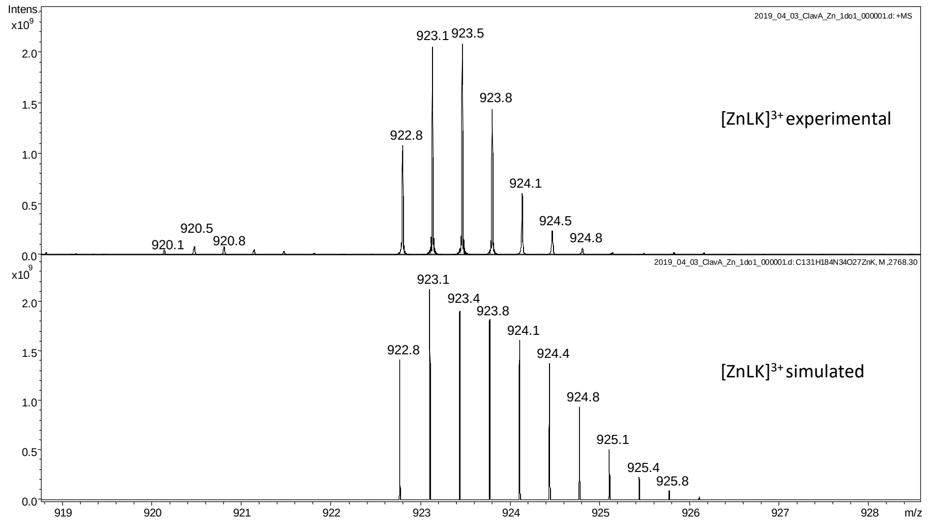

[ZnLK]3+ experimental
[ZnLK]3+ simulated
m/z


B.

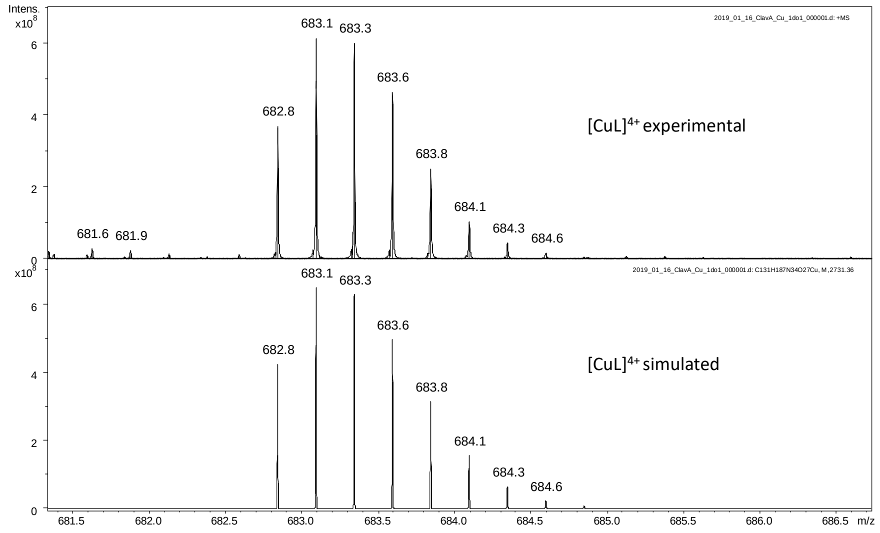

[CuL]4+ experimental
[CuL]4+ simulated
m/z


C.

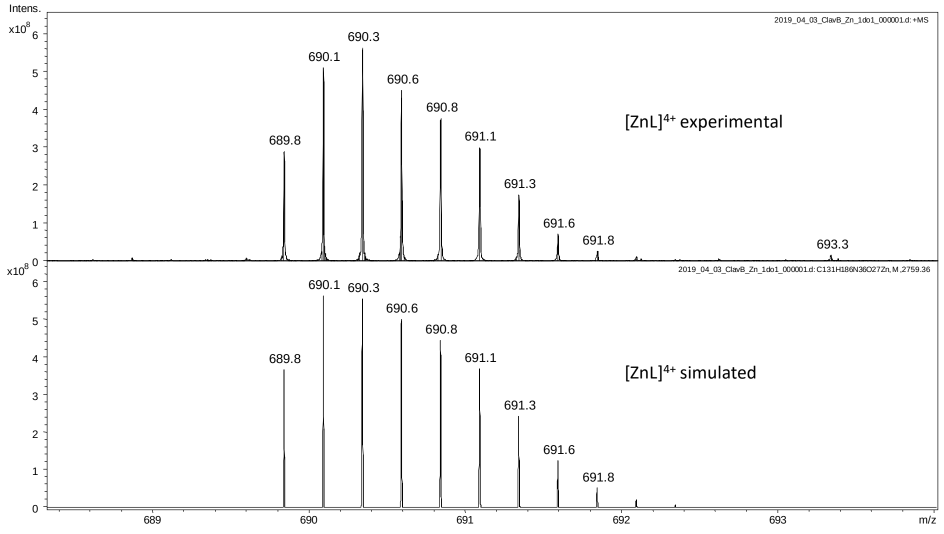

[ZnL]4+ experimental
[ZnL]4+ simulated
m/z

D.

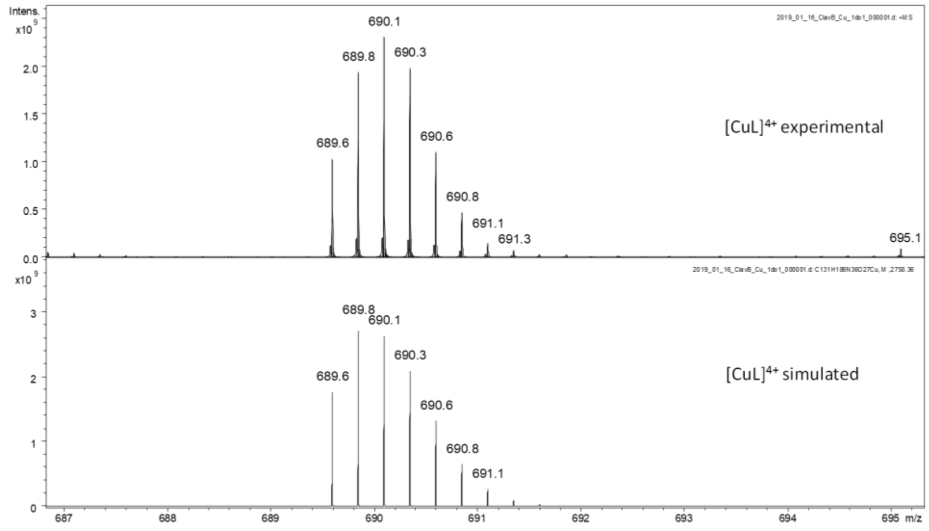
Intens.
x10^9
[CuL]4+ experimental
689.6
689.8
690.1
690.3
690.6
690.8
691.1
691.3
695.1
[CuL]4+ simulated
m/z

E.

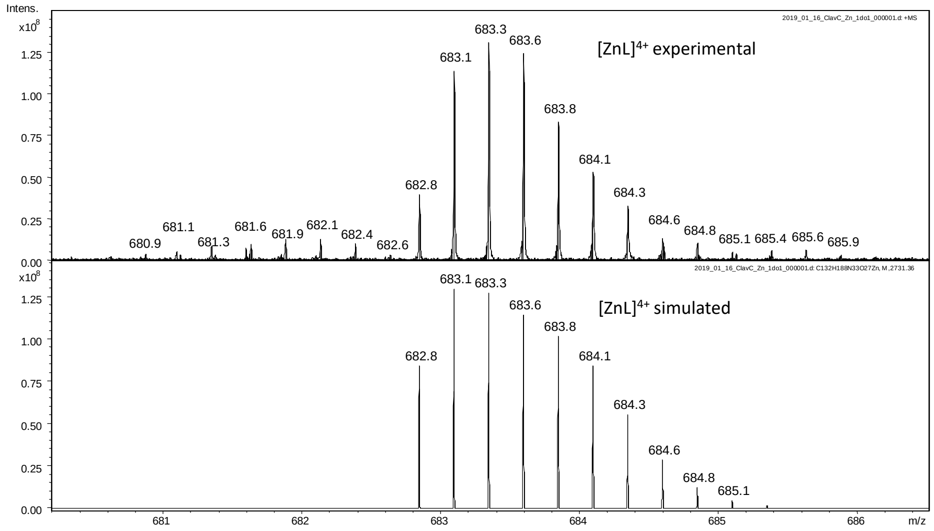
Intens.
x10^8
[ZnL]4+ experimental
683.1
683.3
683.6
683.8
684.1
684.3
[ZnL]4+ simulated
m/z

F.

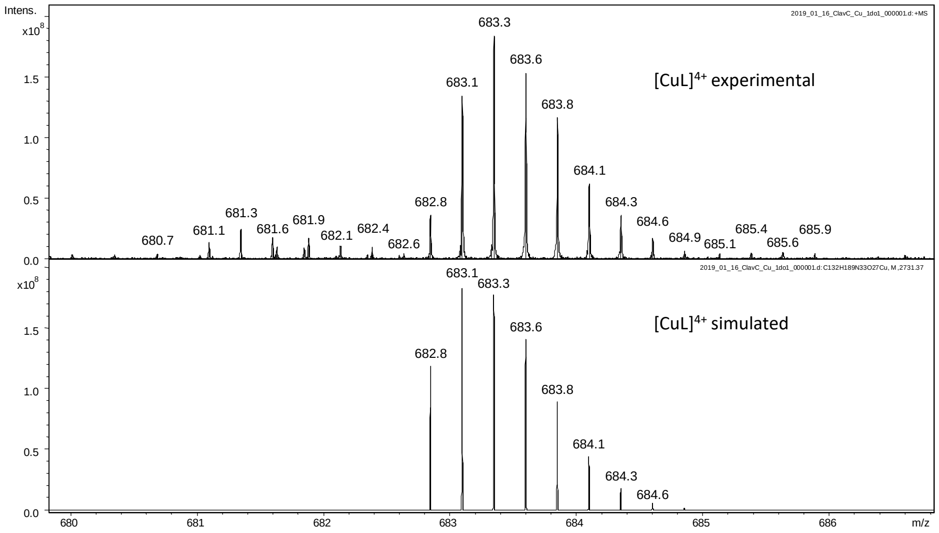
Intens.
x10^8
[CuL]4+ experimental
683.1
683.3
683.6
683.8
684.1
684.3
[CuL]4+ simulated
m/z

G.

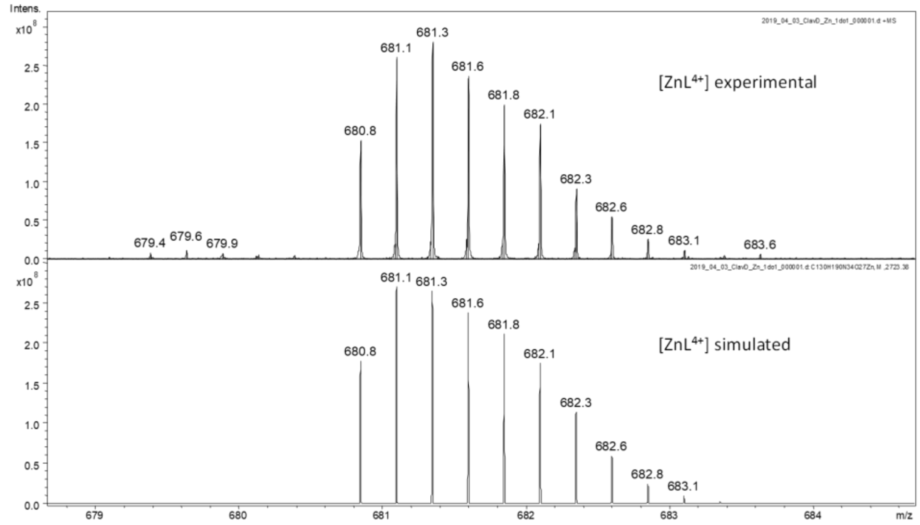

Intens.
x10^8
[ZnL4+] experimental
[ZnL4+] simulated
680.8
681.1
681.3
681.6
681.8
682.1
682.3
682.6
682.8
683.1
683.6
679.4
679.6
679.9
m/z


H.

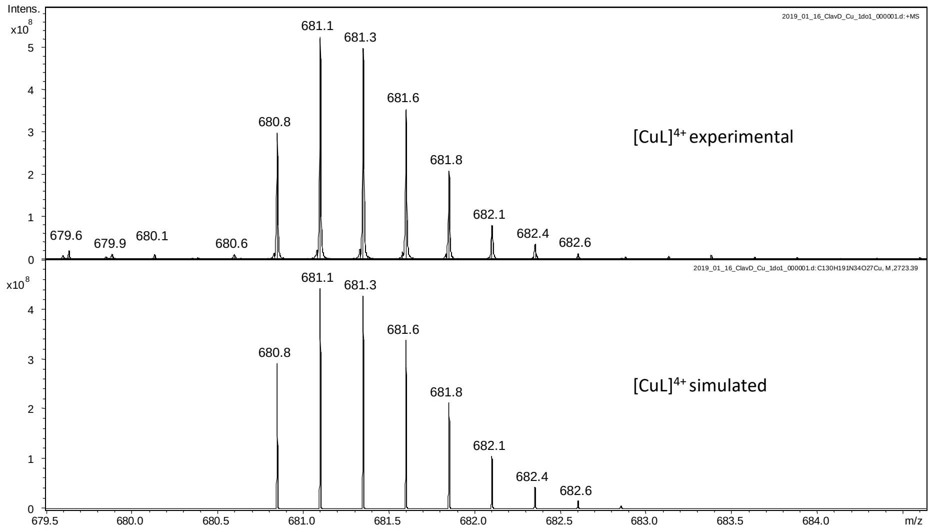

Intens.
x10^8
[CuL]4+ experimental
[CuL]4+ simulated
679.6
679.9
680.1
680.6
680.8
681.1
681.3
681.6
681.8
682.1
682.4
682.6
m/z


I.

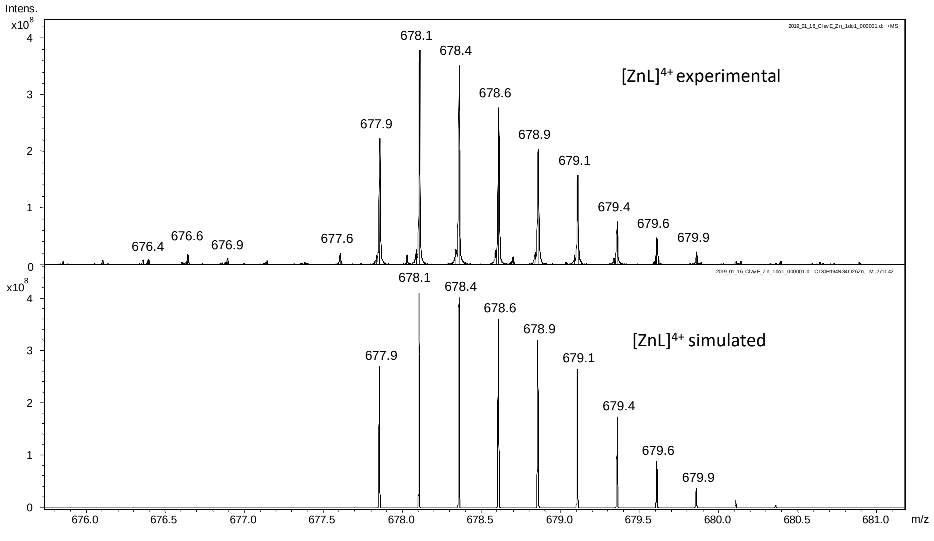

Intens.
x10^8
[ZnL]4+ experimental
[ZnL]4+ simulated
676.4
676.6
676.9
677.6
677.9
678.1
678.4
678.6
678.9
679.1
679.4
679.6
679.9
m/z

J.

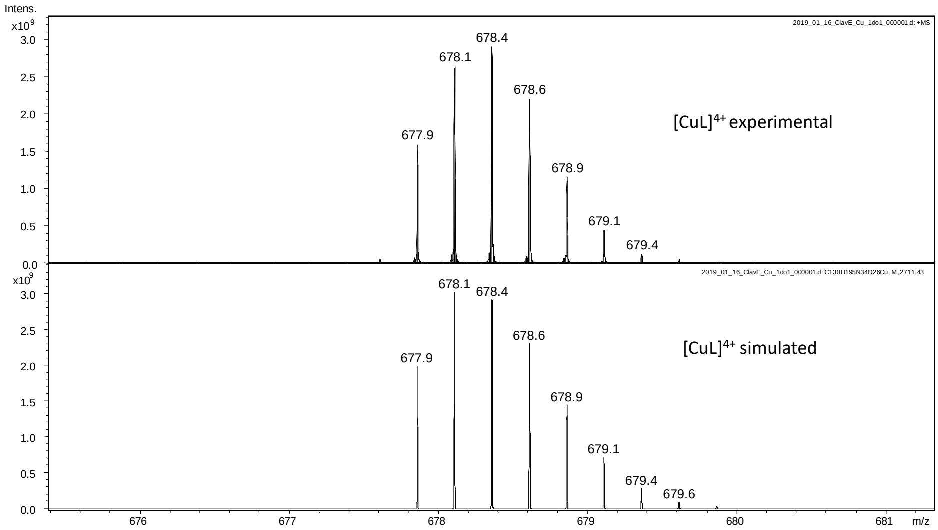


**Fig. S3.** Isotopic distribution of A) Zn(II)-clavanin A; B) Cu(II)-clavanin A; C) Zn(II)-clavanin B; D) Cu(II)-clavanin B; E) Zn(II)-clavanin C; F) Cu(II)-clavanin C; G) Zn(II)-clavanin D; H) Cu(II)-clavanin D; I) Zn(II)-clavanin E; J) Cu(II)-clavanin E; $M^{2+}$:L molar ratio = 1:1, pH = 7.4

## Protonation of the ligands

For each of the studied clavanins, six protonation constants were determined. For clavanin A (VFQFLGKIIHHVGNFVHGFSHVF-COOH), the first four constants come from the deprotonation of four histidine imidazole groups, with pKa values of 5.32, 6.92, 7.63 and 8.09. The next constant is related to the deprotonation of the N-terminal amine group (pKa = 8.88) and the last one, with the pKa value of 10.18 – with the lysine side chain (Table S1).

Clavanin B (VFQFLGRIIHHVGNFVHGFSHVF-COOH) differs from the clavanin A only by the presence of an arginine instead of lysine. The first four constants are related to the deprotonation of imidazole groups of four histidines (pKa = 5.12, 7.09, 7.79, and 8.26); the subsequent pKa values of 9.04 and 11.17 correspond to the deprotonation of N-terminal amine group and the arginine side chain, respectively (Table S1).

In the clavanin C (VFHLLGKIIHHVGNFVYGFSHVF-COOH), protonation constants for the four histidine imidazole groups (pKa values of 5.25, 7.10, 7.57, 8.08), N-terminal amine group (pKa = 8.87), and the arginine side chain (pKa = 10.72) were determined (Table S1).

Clavanin D (AFKLLGRIIHHVGNFVYGFSHVF-COOH), unlike the other clavanins, contains only three histidine residues. Therefore, only the first three constants (pKa values of 5.26, 7.24 and 7.81) are derived from the deprotonated histidine imidazole groups, the next one (pKa = 8.68) comes from deprotonation of the N-terminal amine group, and the last two constants (pKa values of 10.08 and 11.13) are associated with the tyrosine and lysine side chains deprotonation, respectively (Table S1).

In the case of the clavanin E (LFKLLGKIIHHVGNFVHGFSHVF-COOH), the first four constants (pKa values of 5.55, 7.30, 7.82 and 8.29) were assigned to the deprotonated histidine residues. The following two constants with pKa values of 9.19 and 10.77 are related to the deprotonation of the N-terminal amine group and tyrosine, respectively (Table S1).

**Table S1.** Potentiometric data for proton, Zn(II) and Cu(II) complexes with VFQFLGKIIHHVGNFVHGFSHVF-COOH, VFQFLGRIIHHVGNFVHGFSHVF-COOH, VFHLLGKIIHHVGNFVYGFSHVF-COOH, AFKLLGRIIHHVGNFVYGFSHVF-COOH and LFKLLGKIIHHVGNFVHGFSHVF-COOH

| | **Clavanin A VFQFLGKIIHHVGNFVHGFSHVF-COOH** | | **Clavanin B VFQFLGRIIHHVGNFVHGFSHVF-COOH** | | **Clavanin C VFHLLGKIIHHVGNFVYGFSHVF-COOH** | | **Clavanin D AFKLLGRIIHHVGNFVYGFSHVF-COOH** | | **Clavanin E LFKLLGKIIHHVGNFVHGFSHVF-COOH** | |
|---|---|---|---|---|---|---|---|---|---|---|
| **Species** | ***log β*** | **p*K*$_a$** | ***log β*** | **p*K*$_a$** | ***log β*** | **p*K*$_a$** | ***log β*** | **p*K*$_a$** | ***log β*** | **p*K*$_a$** |
| HL | 10.18 (6) | 10.18 (Lys) | 11.17 (1) | 11.17 (Arg) | 10.72 (1) | 10.72 (Tyr) | 11.13 (6) | 11.13 (Lys) | 10.77 (2) | 10.77 (Lys) |
| $H_2L$ | 19.06 (6) | 8.88 (N-t) | 20.21 (2) | 9.04 (N-t) | 19.59 (2) | 8.87 (N-t) | 21.21 (4) | 10.08 (Tyr) | 19.96 (3) | 9.19 (N-t) |
| $H_3L$ | 27.15 (8) | 8.09 (His) | 28.47 (2) | 8.26 (His) | 27.67 (2) | 8.08 (His) | 29.89 (5) | 8.68 (N-t) | 28.25 (3) | 8.29 (His) |
| $H_4L$ | 34.78 (7) | 7.63 (His) | 36.26 (2) | 7.79 (His) | 35.24 (2) | 7.57 (His) | 37.70 (5) | 7.81 (His) | 36.07 (3) | 7.82 (His) |
| $H_5L$ | 41.7 (8) | 6.92 (His) | 43.35 (2) | 7.09 (His) | 42.34 (2) | 7.10 (His) | 44.94 (5) | 7.24 (His) | 43.37 (3) | 7.30 (His) |
| $H_6L$ | 47.02 (10) | 5.32 (His) | 48.47 (2) | 5.12 (His) | 47.59 (2) | 5.25 (His) | 50.20 (5) | 5.26 (His) | 48.92 (4) | 5.55 (His) |
| **Zn(II) complexes** | | | | | | | | | | |
| $ZnH_5L$ | - | - | - | - | - | | 47.63 (12) | | - | |
| $ZnH_4L$ | 37.74 (12) | | 39.23 (7) | | 38.39 | | - | | - | |
| $ZnH_3L$ | 31.14 (3) | 6.59 (His) | 32.61 (2) | 6.62 (His) | - | | 34.16 (3) | | 32.30 (11) | |
| $ZnH_2L$ | 23.99 (2) | 7.15 (His) | 25.24 (2) | 7.37 (His) | 24.25 (10) | | 25.94 (2) | 8.22 (N-t) | 25.12 (5) | 7.18 (His) |
| ZnHL | 15.46 (2) | 8.53 (N-t) | 16.85 (1) | 8.39 (N-t) | - | | - | | 16.50 (6) | 8.62 (N-t) |
| ZnL | 6.10 (7) | 9.36 (Lys) | 6.53 (7) | 10.32 (Arg) | 6.84 (11) | | 6.73 (14) | | 6.73 | 9.77 (Lys) |
| **Cu(II) complexes** | | | | | | | | | | |
| $CuH_5L$ | - | | - | | 45.74 (8) | | 48.56 (3) | | 47.54 (3) | |
| $CuH_4L$ | 40.36 (2) | | 41.57 (3) | | 40.75 (3) | 4.99 | 43.33 (1) | 5.23 (His) | 42.38 (2) | 5.16 (His) |
| $CuH_3L$ | 34.35 (2) | 6.01 (His) | 35.57 (2) | 5.99 (His) | - | | 37.33 (2) | 6.00 (His) | 36.42 (3) | 5.96 (His) |
| $CuH_2L$ | 27.53 (3) | 6.82 (amide) | 28.72 (3) | 6.86 (amide) | 29.28 (3) | | 30.38 (2) | 6.95 (amide) | 29.52 (3) | 6.90 (amide) |
| CuHL | 19.97 (3) | 7.56 (His) | 21.00 (3) | 7.71 (His) | 23.18 (4) | 6.10 (His) | 22.44 (3) | 7.94 (N-t) | 21.80 (3) | 7.72 (His) |
| CuL | 11.61 (3) | 8.36 (N-t) | 12.37 (3) | 8.64 (N-t) | 16.20 (4) | 6.98 (His) | 13.65 (3) | 8.79 (amide) | 12.99 (4) | 8.81 (N-t) |
| $CuH_{-1}L$ | 2.79 (3) | 8.82 (amide) | 2.96 (3) | 9.40 (amide) | 8.11 (6) | 8.09 (His) | 3.73 (3) | 9.92 (amide) | 4.21 (4) | 8.78 (amide) |
| $CuH_{-2}L$ | -6.97 (3) | 9.76 (amide) | -7.17 (3) | 10.14 (amide) | -2.55 (7) | 10.66 (Tyr) | -6.81 (4) | 10.54 (Tyr) | -5.84 (4) | 10.05 (amide) |
| $CuH_{-3}L$ | -17.13 (3) | 10.16 (Lys) | -18.46 (4) | 11.28 (Arg) | | | -18.72 (1) | 11.91 (Lys) | -16.21 (5) | 10.37 (Lys) |

## Zn(II) complexes

Overall stabilities for **Zn(II)-clavanin complexes** are very similar (Table S1, Fig. S4A-E), that is why, below, we discuss only the Zn(II)-clavanin A complex as a representative example.

The first complex observed at acidic pH, $ZnH_4L$, reaches its maximum around pH 6.5; most likely, only one histidine imidazole is involved in the binding of Zn(II) at this pH. In the next form, $ZnH_3L$, (pKa= 6.59) two imidazole groups are involved in coordination. The next deprotonation results in the formation of the $ZnH_2L$ complex, which dominates at around physiological pH; its pKa value (7.15) suggests that the metal is coordinated to three imidazole groups (the pKa of the corresponding imidazole in the free ligand = 7.63). The ZnHL species (pKa = 8.53) most probably come from the deprotonation of the N-terminal amine group, which does not take a part in the coordination. This was directly confirmed for the clavanin D complex, which was chosen as a representative example of Zn(II)-clavanin complexes, due to the facility of signal assignment – all other clavanins have a valine or leucine at their N termini, which heavily overlap with other valines and leucines from the clavanin sequences, while clavanin D has a unique alanine at its N-terminus and therefore it was

relatively easy to show by NMR that the N-terminal $NH_2$ group does not take part in the binding; signals from the N-terminal alanine in clavanin D were unaltered in the Zn(II)-clavanin D spectra at pH 7.4 with respect to those of the free ligand (Fig. S5A). The last, ZnL complex of clavanin A dominates in solution at pH around 9.8. Its pKa value indicates the deprotonation of the lysine side chain, which has no impact on the complex coordination mode (Fig. S4A)

A.

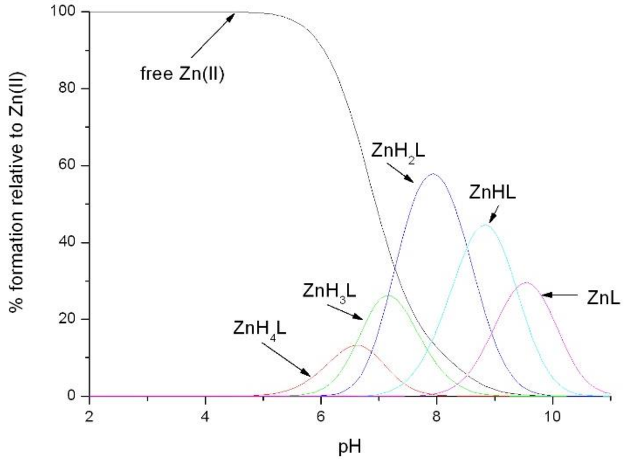


B.

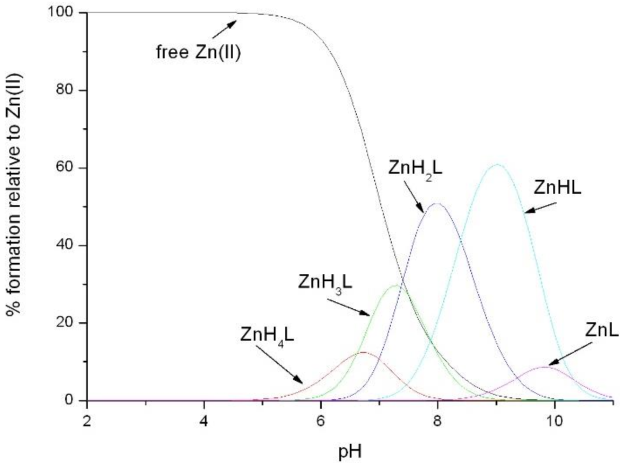


C.

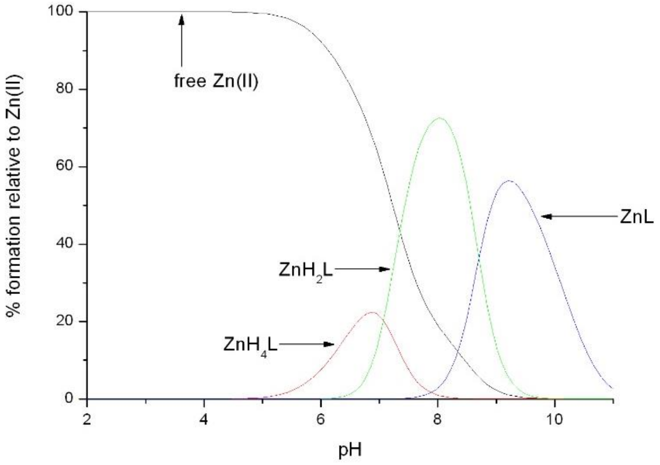

D.

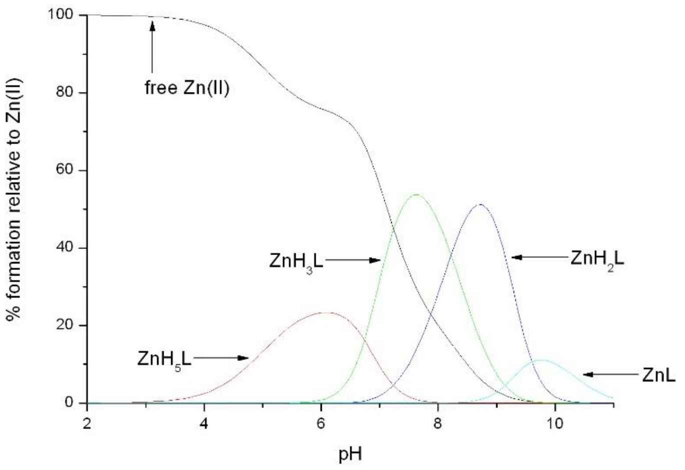


E.

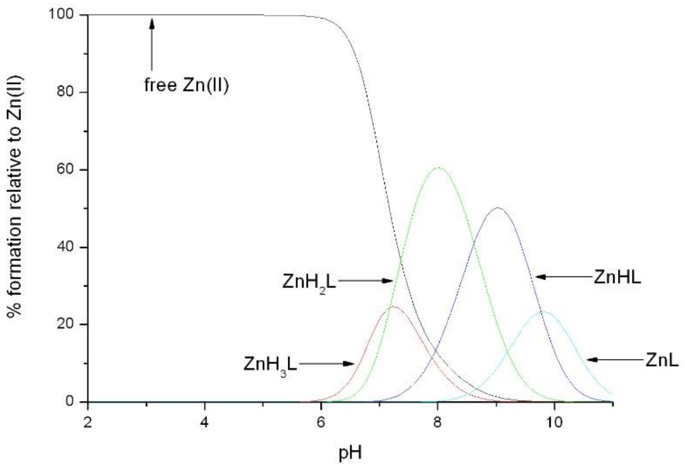


**Fig. S4.** Distribution diagrams for the formation of: A) Zn(II) complex with clavanin A; B) Zn(II) complex with clavanin B; C) Zn(II) complex with clavanin C; D) Zn(II) complex with clavanin D; E) Zn(II) complex with clavanin E; T=298 K, I= 40 mM SDS, $[M^{2+}]=0.5\cdot10^{-3}$ M; $M^{2+}$:L molar ratio = 1:1

A.

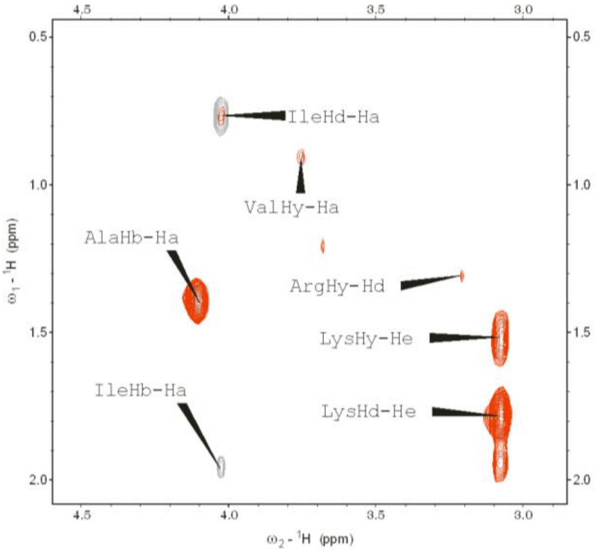

B.

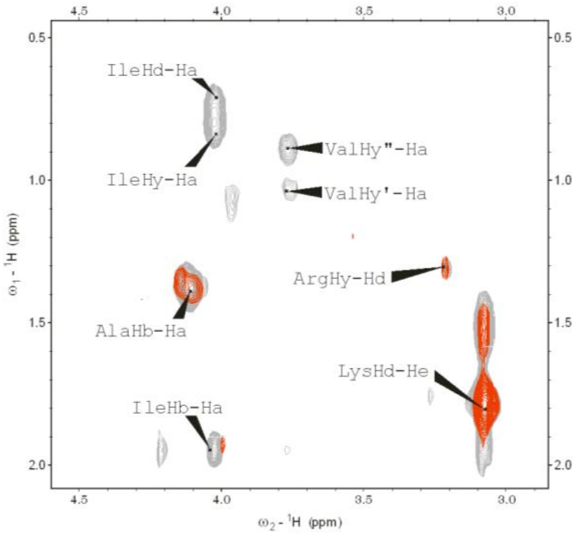


**Fig. S5.** 1H–1H TOCSY NMR spectra of a fragment of the clavanin D (gray) and the complex (red) with Zn(II) (A) or Cu(II) (B); [clavanin D] = 3 mM; Zn(II):L molar ratio 1:1, Cu(II):L molar ratio 1:0.2; pH = 3, T = 298 K, I = 40 mM dSDS in 90 % $H_2O$ and 10 % $D_2O$ solution.

**Table S2.** Metal – ligand distances in angstroms for clavanin A, B, C, D, E and Zn(II) complexes.

| | clavanin A | clavanin B | clavanin C | clavanin D | clavanin E |
|---|---|---|---|---|---|
| **H10 (imidazole)** | 2.106 | 2.107 | 2.170 | 2.164 | 2.192 |
| **H11 (imidazole)** | 2.017 | 2.008 | 2.196 | 2.193 | 2.070 |
| **H17 (imidazole)** | 2.038 | 2.016 | | | 2.054 |
| **H21 (imidazole)** | | | 2.199 | 2.030 | |

## Cu(II) complexes

Similarly to the Zn(II) complexes, all five examined clavanins form mononuclear complexes with Cu(II), which also was proved by ESI-MS spectra. The stability constants for Cu(II) complexes are presented in Table S1. In pH range 2-11, Cu(II)-clavanin A forms 8 complex species (Fig. S6A). Copper(II) starts to coordinate at pH above 4, forming the $CuH_4L$ complex, which reaches its maximum at pH 5.5. In this complex, Cu(II) is bound to two imidazole nitrogens ($2N_{im}$) from two histidine side chains, as suggested by the UV-Vis maximum at around 680 nm (Fig. S9A). At pH 6.5, a maximum of the $CuH_3L$ form is observed. Its pKa value of 6.01 and a blue shift of the UV-Vis maximum confirms that a third histidine imidazole group is involved in Cu(II) coordination. After the next deprotonation, the $CuH_2L$ complex appears (maximum at pH 7.2, in which the first amide group takes part in the binding (pKa = 6.82)). Coordination of the amide group is additionally confirmed by a specific d-d band which starts to appear in the CD spectra (Fig. S8A).[22] At pH 8, the maximum of the CuHL form is observed, in which the deprotonation of a non-binding imidazole group occurs (the pKa of this residue in the free ligand = 8.09 and in the complex – 7.56; small difference between those values suggests the non-bonding character). The following pKa value (8.36) corresponds to the deprotonation of the non-coordinating N-terminal amine group (CuL) – pKa in the free ligand equals 8.88. The lack of participation of the N-terminal $NH_2$ group in metal binding was additionally confirmed by NMR, where correlations of all N-terminal alanine protons in the Cu(II)-clavanin D complex remain unchanged with respect to those of the free clavanin D (Fig. S5B). Clavanin D was used as a model (as in the case of Zn(II) complexes), because of the presence of alanine on the N-terminus,

which was a unique amino acid in all peptide's sequence. Other peptides contain valine or leucine in this position, which were also repeated in the peptide chain, thus changes caused by the effect of metal ion on the N-terminus could have been unnoticed. Since the Cu(II)-complex forms and formation constants for clavanin A, B, D and E are very similar, this allows us to conclude a similar coordination pattern in all mentioned species (Fig. S6 B, D and E).

The last complex, $CuH_{-3}L$, refers to the deprotonation of the lysine side chain, which does not participate in Cu(II) binding (pKa values for the protonation and stability constants equal 10.18 and 10.16 respectively).

In the case of Cu(II)-clavanin B, the coordination mode is analogous to the Cu(II)-clavanin A one, with deprotonation of arginine (pKa = 11.28) instead of lysine (Fig. S6B, S8B, S9B). Clavanin E also behaves analogously to clavanin A, showing almost identical spectroscopic data for analogous Cu(II) complexes with similar stability (Fig. S6E, S8E, S9E).

Clavanin C is the only clavanin with a histidine in the third position of the peptide sequence. This pattern ($NH_2$-Xaa-Yaa-His), also called the ATCUN motif, allows to form very stable, square-planar complexes with Cu(II) and Ni(II) [23]. In the case of clavanin C, in the $CuH_2L$ complex, with a maximum at pH 6, two amides, the histidine imidazole and the N-terminal amine group simultaneously coordinate to the Cu(II) ion, resulting in a ($N_{im}$, $NH_2$, $2N_-$) binding mode. For the UV-Vis measurements in pH range 2-5, no clear peaks between 450-700 nm were detected, but for samples measured at pH above 6, a signal with $\lambda_{max}$= 510 nm was observed, which confirms participation of four nitrogens in Cu(II) coordination (Fig. S9C).[22] The situation, when all four nitrogens bind Cu(II) rapidly is opposite to the rest of the clavanins, where the $\lambda_{max}$ of the signals was moving towards the shorter wavelengths together with increasing pH (Fig. S9A, S9B, S9D, S9E). The sudden formation of a square – planar complex at pH between 5 and 6 was also observed in the CD spectra, as a rapid appearance of two peaks with $\lambda_{max}$ = 490 nm and $\lambda_{min}$ = 555 nm at pH above 5 (Fig. S8C).

Stability constants with pKa values of 6.10, 6.98, 8.09 and 10.66 correspond to the deprotonation of three imidazole groups and tyrosine side chain, respectively. Those groups do not participate in Cu(II) coordination, what is proved by lack of spectroscopic changes and minor differences between protonation constants of the free ligand and the complex stability constants (7.10, 7.57, 8.08 and 10.72 respectively) (Fig. S6C, S8C, S9C).

In clavanin D, the first three acidic species ($CuH_5L$, $CuH_4L$, and $CuH_3L$; Fig. S6D) are related to the coordination of the three histidine side chains; the UV-Vis maximum of these complexes undergoes a continuous blue shift from 720 to 623 nm (Fig. S9D). The next deprotonation leads to the $CuH_2L$ complex, with maximal abundance at pH around 7.2. In these species, an additional amide nitrogen participates in Cu(II) binding, what is suggested by the CD spectra, which show d-d bands characteristic for amide-involving, square planar complexes (Fig. S8D).[22] The subsequent complex form, CuHL with a pKa value of 7.94 is the most abundant at pH 8.3 and corresponds to the deprotonation of the non-bonding N-terminal amine group – the difference between the pKa values for protonation (8.68) and stability constants (7.94) is not large, what suggests a non-bonding character. Furthermore, no pronounced spectral changes are observed with respect to the previous complex (Fig. S8D, S9D). The loss of a further proton leads to the CuL species (pKa value = 8.79), which shows its maximal level at pH above 9 and is associated with another amide nitrogen coordination – the UV-Vis $\lambda_{max}$ moves towards shorter wavelengths, from 610 nm to 530 nm (registered for the pH 10; Fig. S9D). The $CuH_{-1}L$ complex form (pKa = 9.92) achieves its maximum at pH above 10. In this complex Cu(II) is bound to three amide nitrogens, what is confirmed by the slight $\lambda_{max}$ to 520 nm (Fig. S9D). Also in the CD spectra the square-planar complex formation can be observed – at pH 11 two well-marked peaks ($\lambda_{min}$ = 500 nm and $\lambda_{max}$ = 640 nm) are present (Fig. S8D). For the $CuH_{-2}L$ complex, deprotonation of the tyrosine side chain can be observed – the differences between the protonation (10.08) and stability constants (10.54) are minor, what suggests the non-bonding character. The last form, observed at basic pH ($CuH_{-3}L$, pKa = 11.91) corresponds to the deprotonation of the non-binding lysine side chain; the non-bonding character is confirmed by the comparison of the protonation and stability constants (11.13 and 11.91 respectively).

A.

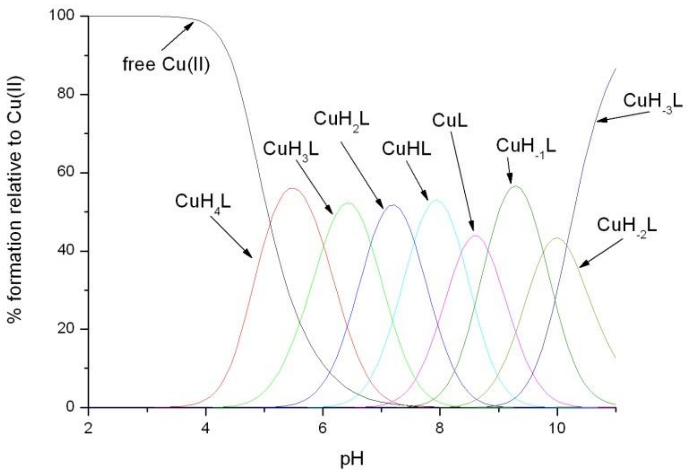

free Cu(II)
CuH$_{-3}$L
CuH$_2$L
CuL
CuH$_3$L
CuHL
CuH$_{-1}$L
CuH$_4$L
CuH$_{-2}$L
% formation relative to Cu(II)
pH


B.

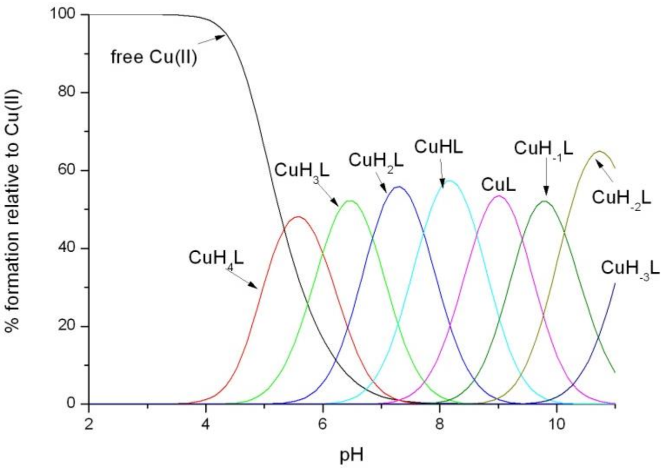

free Cu(II)
CuH$_3$L
CuH$_2$L
CuHL
CuH$_{-1}$L
CuL
CuH$_{-2}$L
CuH$_4$L
CuH$_{-3}$L
% formation relative to Cu(II)
pH


C.

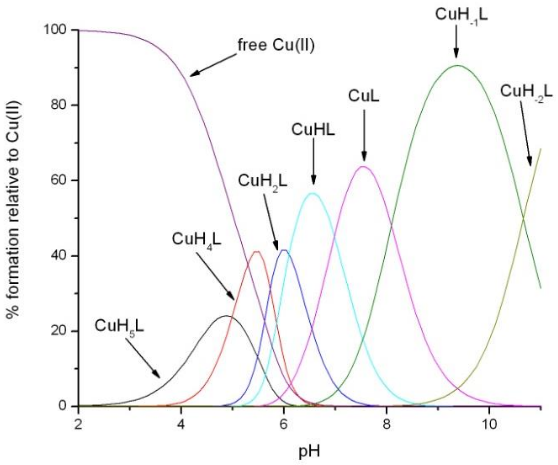

CuH$_{-1}$L
free Cu(II)
CuL
CuH$_{-2}$L
CuHL
CuH$_2$L
CuH$_4$L
CuH$_5$L
% formation relative to Cu(II)
pH

D.

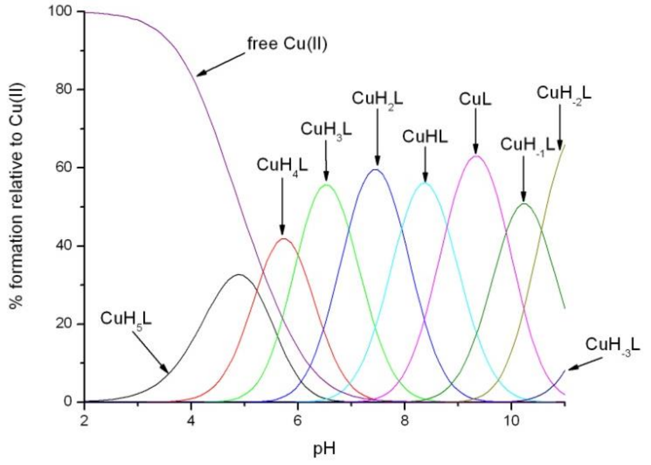


E.

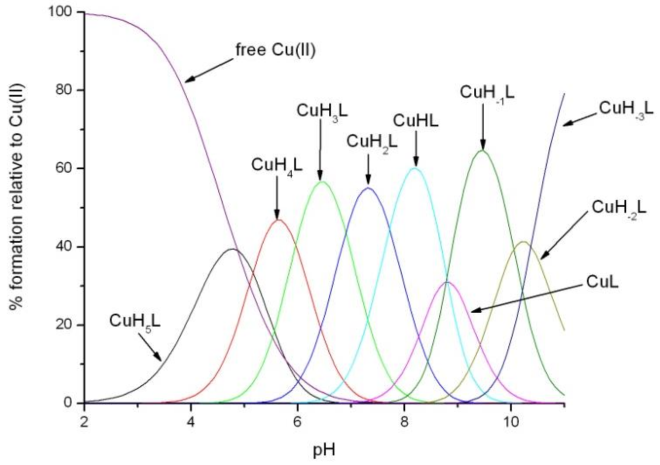


**Fig. S6.** Distribution diagrams for the formation of: A) Cu(II) complex with clavanin A; B) Cu(II) complex with clavanin B; C) Cu(II) complex with clavanin C; D) Cu(II) complex with clavanin D; E) Cu(II) complex with clavanin E; T=298 K, I= 40 mM SDS, $[M^{2+}]=0.5\cdot10^{-3}$ M; $M^{2+}$:L molar ratio = 1:1

**Table S3**. Metal – ligand distances in angstroms for clavanin A, B, C, D, E and Cu(II) complexes.

| | clavanin A | clavanin B | clavanin C | clavanin D | clavanin E |
|---|---|---|---|---|---|
| **H10 (imidazole)** | 1.987 | 2.000 | | 2.046 | 1.982 |
| **H11 (imidazole)** | 2.003 | 2.063 | | 2.047 | 1.981 |
| **H17 (imidazole)** | 1.952 | 2.000 | | | 2.161 |
| **H21 (imidazole)** | | | | 1.986 | |
| **H17 (amide)** | 1.891 | 1.976 | | | 1.987 |
| **H3 (imidazole)** | | | 2.081 | | |
| **V1 (N-terminus)** | | | 2.159 | | |
| **F2 (amide)** | | | 2.066 | | |
| **H3 (amide)** | | | 2.117 | | |
| **H11 (amide)** | | | | 2.016 | |

A.

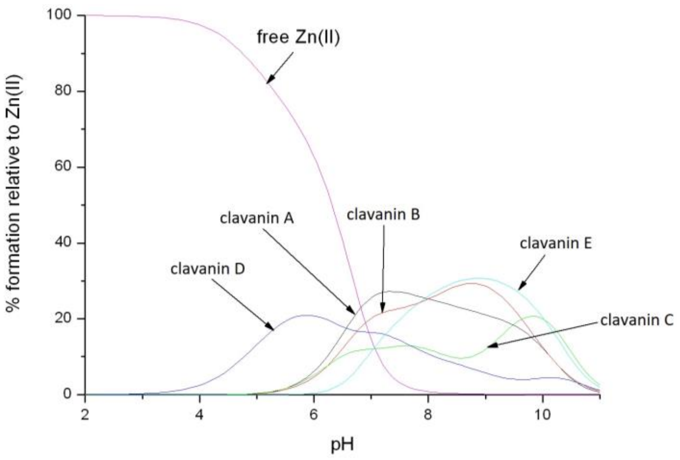


B.

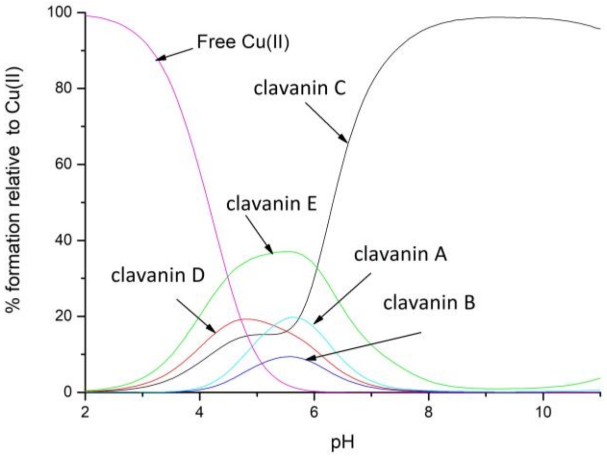


**Fig. S7** Competition plot between clavanin A, B, C, D, E and Zn(II) (A) or Cu(II) (B), describing complex formation at different pH values in a hypothetical situation, in which equimolar amounts of the six reagents are mixed.

A.

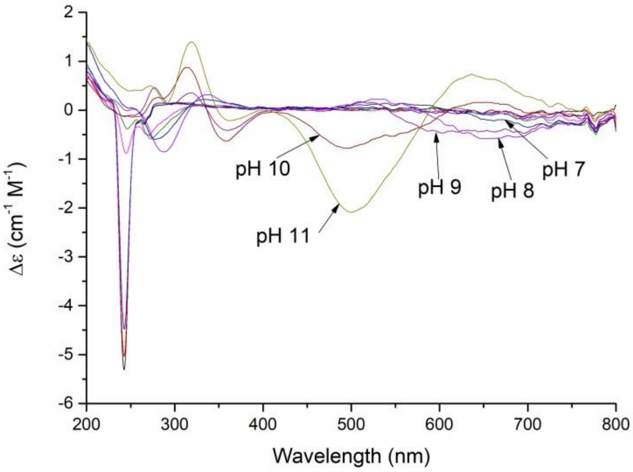
pH 10
pH 11
pH 9
pH 8
pH 7
Δε (cm⁻¹ M⁻¹)
Wavelength (nm)

B.

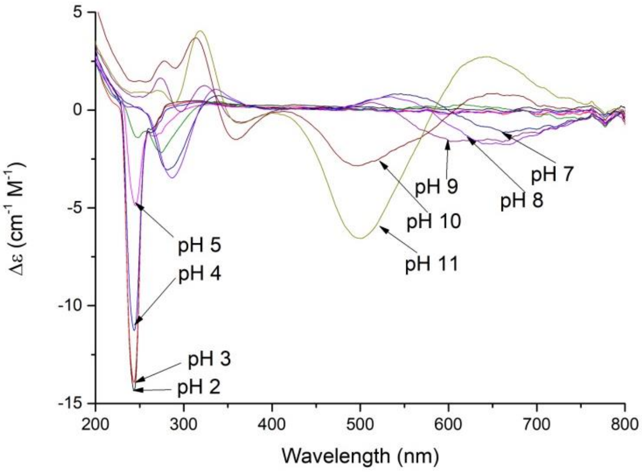
pH 5
pH 4
pH 3
pH 2
pH 9
pH 10
pH 11
pH 8
pH 7
Δε (cm⁻¹ M⁻¹)
Wavelength (nm)

C.

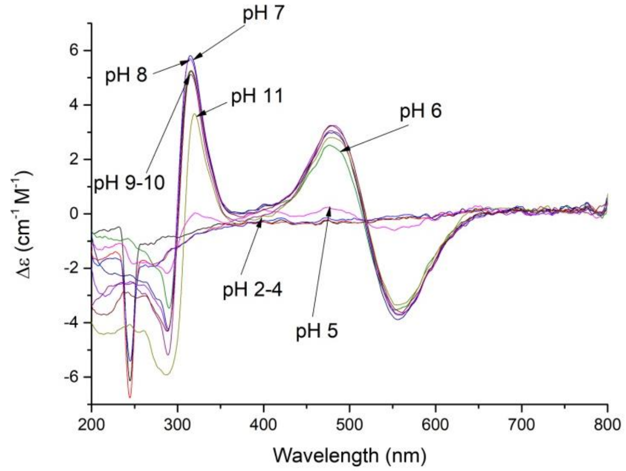
pH 7
pH 8
pH 11
pH 6
pH 9-10
pH 2-4
pH 5
Δε (cm⁻¹ M⁻¹)
Wavelength (nm)

D.

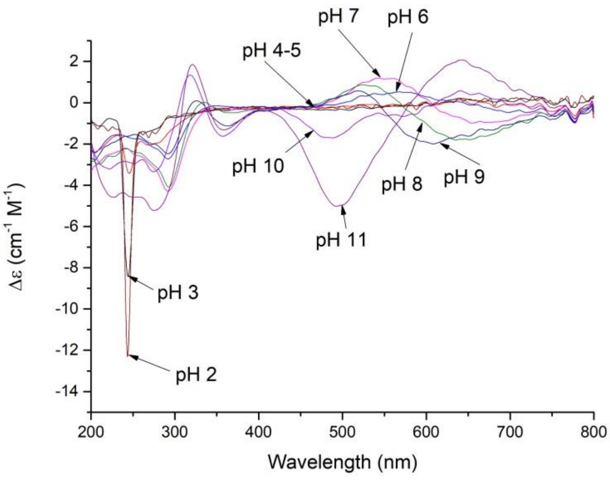


E.

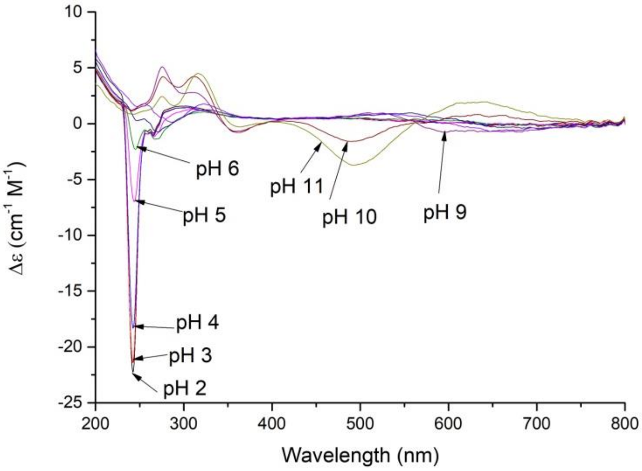


**Fig. S8.** CD spectra of Cu(II) complexes with: A) clavanin A; B) clavanin B; C) clavanin C; D) clavanin D; E) clavanin E; in pH range 2-11. Conditions: T = 298 K, I = 40 mM SDS, [Cu(II)] = [clavanin A] = [clavanin B] = [clavanin C] = [clavanin D] = [clavanin E] = 0.001 M.

A.

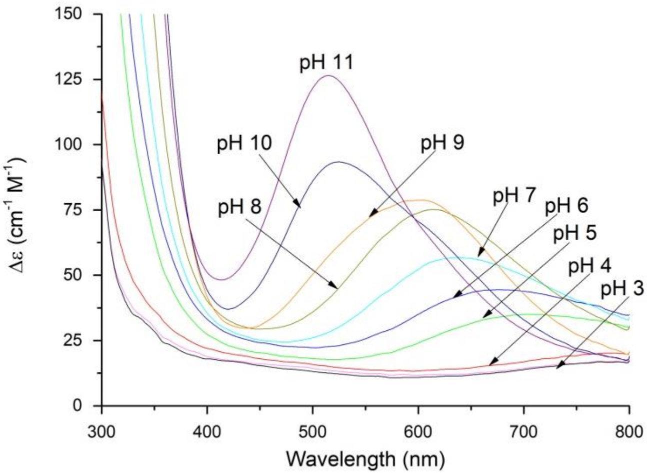

B.

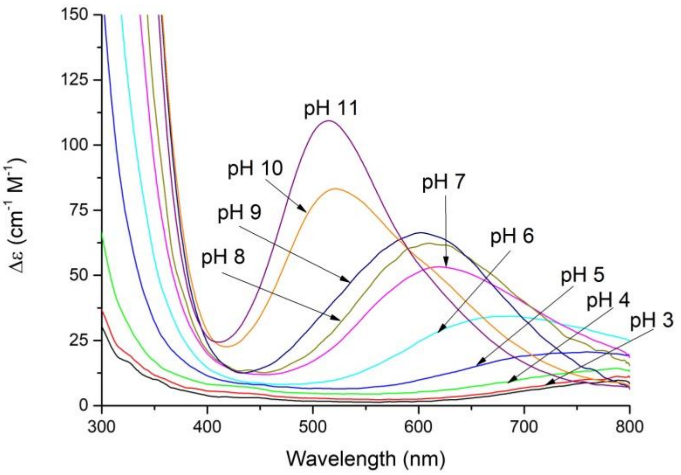

150
125
100
75
50
25
0
300
400
500
600
700
800
Δε (cm-1 M-1)
Wavelength (nm)
pH 11
pH 10
pH 9
pH 8
pH 7
pH 6
pH 5
pH 4
pH 3


C.

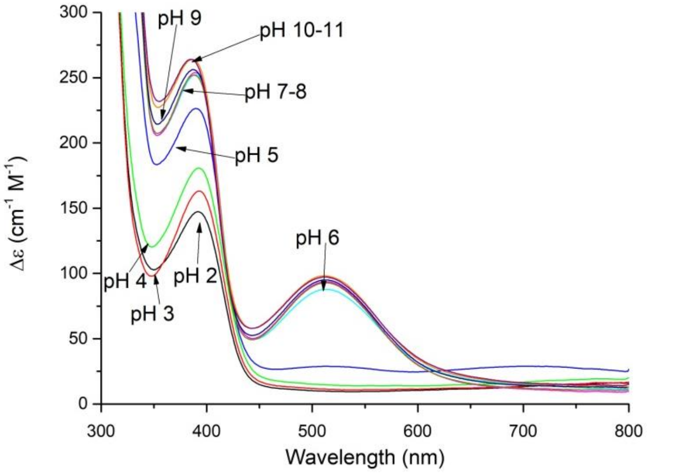

300
250
200
150
100
50
0
300
400
500
600
700
800
Δε (cm-1 M-1)
Wavelength (nm)
pH 9
pH 10-11
pH 7-8
pH 5
pH 6
pH 4
pH 3
pH 2


D.

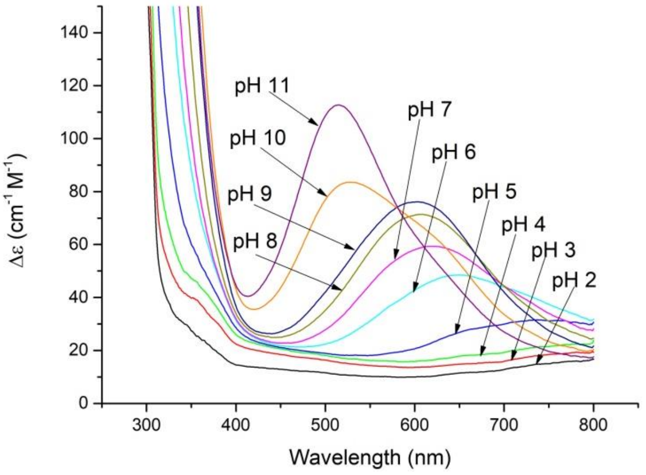

140
120
100
80
60
40
20
0
300
400
500
600
700
800
Δε (cm-1 M-1)
Wavelength (nm)
pH 11
pH 10
pH 9
pH 8
pH 7
pH 6
pH 5
pH 4
pH 3
pH 2

E.

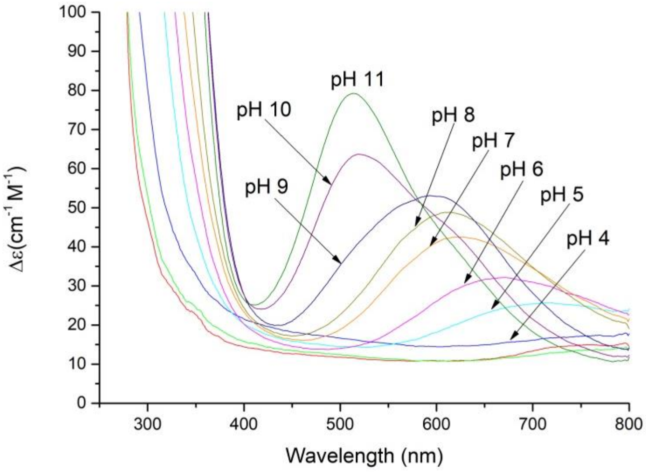


**Fig. S9.** UV-Vis spectra of Cu(II) complexes with: A) clavanin A; B) clavanin B; C) clavanin C; D) clavanin D; E) clavanin E; in pH range 2-11. Conditions: T = 298 K, I = 40 mM SDS, [Cu(II)] = [clavanin A] = [clavanin B] = [clavanin C] = [clavanin D] = [clavanin E] = 0.001 M.

**Table S4**. In vitro antibacterial activity of clavanins A, B, C, D and E determined as a minimal inhibitory concentration (MIC) (µg/mL); n/d, not determined. Experiments were performed for all compounds and their copper(II) and zinc(II) complexes according to the ISO 20776-1:2019[16] and ISO 16256:2012[17]. Colors have been added to improve readability. No MIC value was determined for *Pseudomonas aeruginosa* ATCC 27853. No MBC/MFC activity was observed after performing modified Richard's method.**[19,20]** Bolded values represent concentrations lower than or equal to established by EUCAST breakpoints[22] for selected antimicrobial agents characteristic for given bacterial families.

| Strain | *Escherichia coli (-) ATCC 25922* | *Enterococcus faecalis (+) ATCC 29212* | *Staphylococcus aureus (+) ATCC 43300* | *Candida albicans* ATCC 10231 |
|---|---|---|---|---|
| | MIC (µg/mL) | MIC (µg/mL) | MIC (µg/mL) | MIC (µg/mL) |
| **Clavanin A** | n/d | 256 | n/d | 128 |
| **Cu(II)-Clavanin A** | n/d | n/d | n/d | 128 |
| **Zn(II)-Clavanin A** | 256 | 256 | n/d | 64 |
| **Clavanin B** | 256 | 256 | n/d | 64 |
| **Cu(II)-Clavanin B** | 256 | **8** | n/d | 64 |
| **Zn(II)-Clavanin B** | n/d | **8** | n/d | 128 |
| **Clavanin C** | n/d | 128 | 128 | 64 |
| **Cu(II)-Clavanin C** | 128 | 256 | 128 | 64 |
| **Zn(II)-Clavanin C** | **16** | **64** | **16** | 16 |
| **Clavanin D** | **64** | 128 | 128 | 16 |
| **Cu(II)-Clavanin D** | 256 | 128 | 128 | 32 |
| **Zn(II)-Clavanin D** | 256 | 128 | 128 | 64 |
| **Clavanin E** | n/d | n/d | n/d | 32 |
| **Cu(II)-Clavanin E** | 256 | n/d | n/d | 32 |
| **Zn(II)-Clavanin E** | n/d | n/d | 64 | 16 |

**Table S5.** Examples of MIC breakpoints values from EUCAST/2021/01/01 for bacteria.[22]

| ***Enterobacterales* (for *E. coli*)** | MIC breakpoints (µg/mL) | | ***Enterococcus* spp. (for *E. faecalis*)** | MIC breakpoints (µg/mL) | | ***Staphylococcus* spp. (for *S. aureus*)** | MIC breakpoints (µg/mL) | |
|---|---|---|---|---|---|---|---|---|
| | S≤ | R> | | S≤ | R> | | S≤ | R> |
| **Amoxicillin-clavulanic acid** | 32 | 32 | **Ampicillin** | 4 | 8 | **Amikacin** | 8 | 8 |
| **Piperacillin** | 8 | 8 | **Ampicillin-sulbactam** | 4 | 8 | **Chloramphenicol** | 8 | 8 |
| **Piperacillin-tazobactam** | 8 | 8 | **Amoxicillin** | 4 | 8 | **Fosfomycin *iv*** | 32 | 32 |
| **Ticarcillin** | 8 | 16 | **Amoxicillin-clavulanic acid** | 4 | 8 | **Nitrofurantoin** | 64 | 64 |
| **Ticarcillin-clavulanic acid** | 8 | 16 | **Nitrofurantoin** | 64 | 64 | | | |
| **Cefadroxil** | 16 | 16 | | | | | | |
| **Cefalexin** | 16 | 16 | | | | | | |
| **Fosfomycin *iv*** | 32 | 32 | | | | | | |
| **Fosfomycin oral** | 32 | 32 | | | | | | |
| **Nitrofurantoin** | 64 | 64 | | | | | | |
| **Nitroxoline** | 16 | 16 | | | | | | |

**Table S6.** *In vitro* antibacterial activity of clavanins against clinical strains of indicated species determined as a minimal inhibitory concentration (MIC) (µg/mL); n/d, not determined. No antimicrobial activity was determined for tested clinical strains of *Staphylococcus aureus*.

| | | | *Escherichia coli (-)* | | | *Enterococcus faecalis (+)* |
|---|---|---|---|---|---|---|
| | *HD41* | *HD84* | *P43* | *P47* | | *HD30* |
| | MIC (µg/mL) | MIC (µg/mL) | MIC (µg/mL) | MIC (µg/mL) | | MIC (µg/mL) |
| **Zn(II)-Clavanin C** | 128 | 64 | n/d | 64 | **Cu(II)-Clavanin B** | 128 |
| **Clavanin D** | 256 | 256 | 256 | n/d | **Zn(II)-Clavanin B** | 128 |
| | | | | | **Zn(II)-Clavanin C** | 64 |

## *In vitro* cytotoxicity studies

After MIC determination, the cytotoxic effect of clavanins A, B, C, D and E, as well as their complexes with zinc(II) and copper(II) ions in the concentration values where MIC was observed were checked. The NR cytotoxicity assay was performed using RPTEC cell line from ECACC collection. This method allows to estimate the number of viable cells using their ability to incorporate and bind neutral red dye in lysosomes.[21] In the Table S7 all data have been collected. For clavanins or clavanin-metal ion systems where intrinsic MIC values in comparison with commonly used antimicrobial drugs bolded type was adopted. It must be pointed out that for all investigated compounds high viability was presented. Thus, all clavanins and clavanin-metal ion systems, where enhancement of antimicrobial activity is seen, better than drugs using in infections treatment against specific strain, can be a good starting point for further investigations resulting new potential drug discovery and development.

**Table S7.** Cell viability using Neutral red uptake assay (NR) after 24 and 48 h incubation with clavanins and clavanin-metal ion systems. Used compounds and their concentration were selected after antimicrobial assay.

| Compound | Concentration [ug/mL] | Incubation time | |
|---|---|---|---|
| | | 48 h | 72 h |
| | | Viability [%] | |
| Clavanin A | 256 | 96 | 103 |
| | 128 | 95 | 103 |
| Cu(II)-Clavanin A | 128 | 98 | 102 |
| Zn(II)-Clavanin A | 256 | 104 | 113 |
| | 64 | 103 | 105 |
| Clavanin B | 256 | 102 | 106 |
| | 64 | 92 | 105 |
| **Cu(II)-Clavanin B** | 256 | 104 | 105 |
| | 64 | 104 | 91 |
| | **8** | **105** | **95** |
| **Zn(II)-Clavanin B** | 128 | 102 | 90 |
| | **8** | **98** | **88** |
| Clavanin C | 128 | 53 | 59 |
| | 64 | 83 | 85 |
| Cu(II)-Clavanin C | 128 | 70 | 77 |
| | 64 | 66 | 77 |
| **Zn(II)-Clavanin C** | **64** | **100** | **91** |
| | 32 | 100 | 89 |
| | **16** | **99** | **89** |
| **Clavanin D** | 128 | 78 | 88 |
| | **64** | **98** | **98** |
| | 16 | 100 | 100 |
| Cu(II)-Clavanin D | 256 | 100 | 103 |
| | 128 | 91 | 92 |
| | 32 | 86 | 89 |
| Zn(II)-Clavanin D | 256 | 83 | 89 |
| | 128 | 85 | 86 |
| | 64 | 84 | 88 |
| Clavanin E | 32 | 76 | 97 |
| Cu(II)-Clavanin E | 256 | 101 | 100 |
| | 32 | 98 | 99 |
| Zn(II)-Clavanin E | 16 | 97 | 96 |

## Membrane disrupting ability

To check the membrane disrupting ability of clavanins and their metal complexes, liposome leakage experiments were carried out. Liposomes were filled with 6-carboxyfluorescein. When damaged with the given clavanin or its complex, liposomes were releasing the fluorescent dye, which was detected spectrofluorometrically (Fig. S10A-F); averaged results from four measurements (arithmetic average) are presented).

In general, zinc(II) complexes show very low to no membrane disrupting ability, while copper(II) complexes slightly decrease the activity in comparison to the ligand. Cu(II)-clavanin E is an exception to this trend because a complex is more active than ligand.

Among all investigated clavanins, clavanin E induced the highest dye leakage (53.4 %) after 15 minutes. It was around 8 % more than clavanin D (45.9 %), and 30 % more than clavanin B (22.5 %). The amounts of 6-carboxyfluorescein released by clavanins A and C were similar (10.8 % and 9.1 % respectively). Those results can confirm the hypothesis, that membrane disruption is not clavanins' main target.[23]

A.

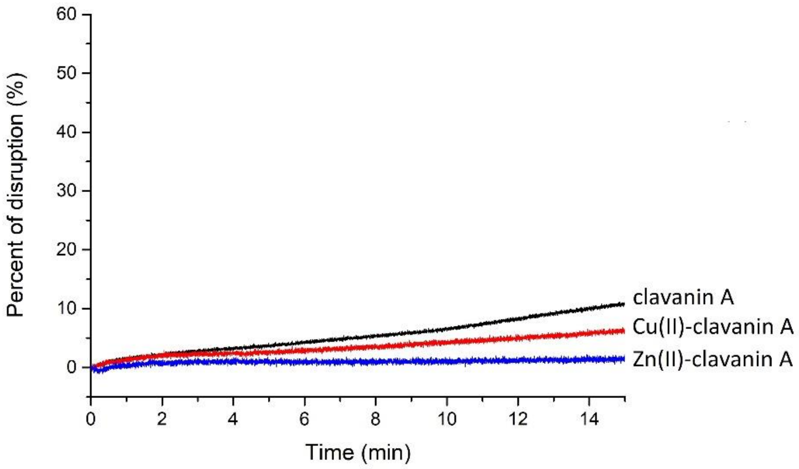


B.

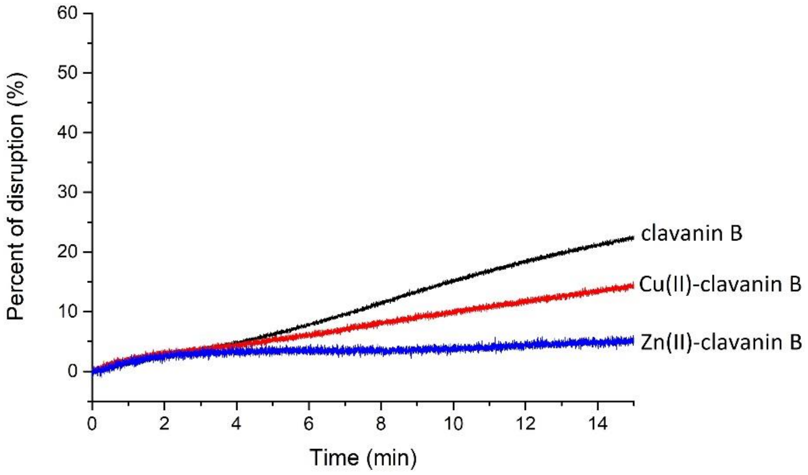

C.

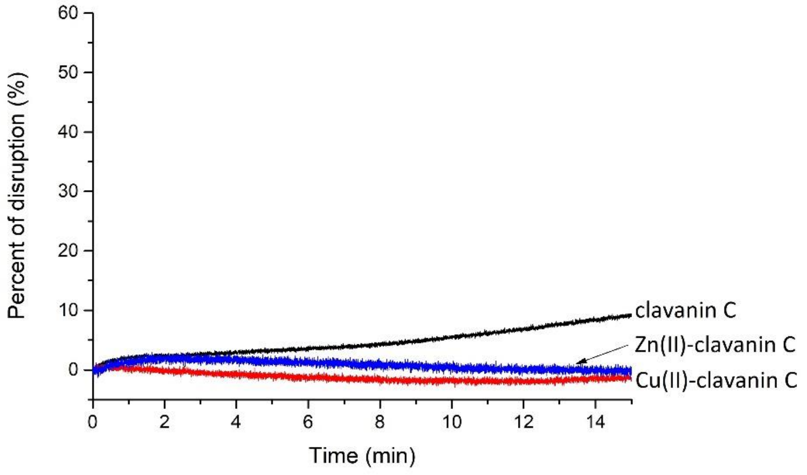

Percent of disruption (%)
60
50
40
30
20
10
0
0
2
4
6
8
10
12
14
Time (min)
clavanin C
Zn(II)-clavanin C
Cu(II)-clavanin C


D.

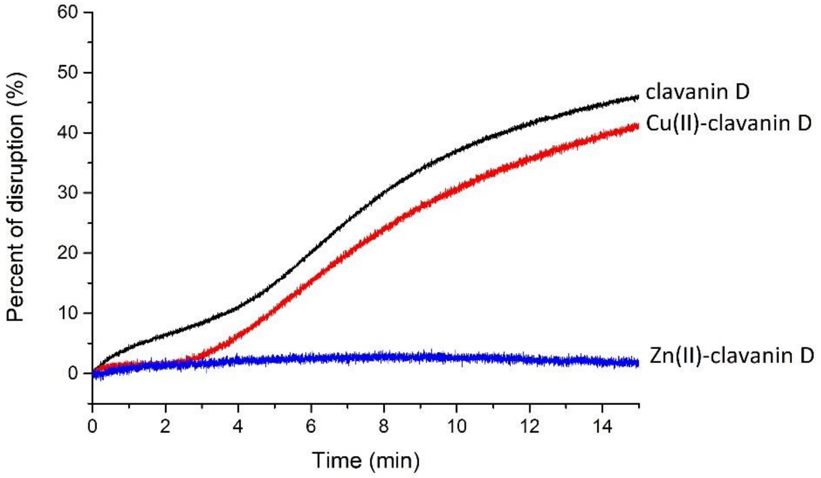

Percent of disruption (%)
60
50
40
30
20
10
0
0
2
4
6
8
10
12
14
Time (min)
clavanin D
Cu(II)-clavanin D
Zn(II)-clavanin D


E.

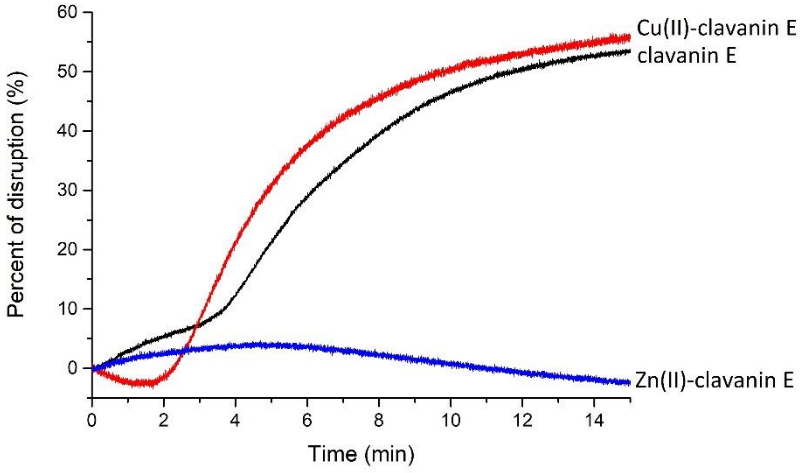

Percent of disruption (%)
60
50
40
30
20
10
0
0
2
4
6
8
10
12
14
Time (min)
Cu(II)-clavanin E
clavanin E
Zn(II)-clavanin E

F.

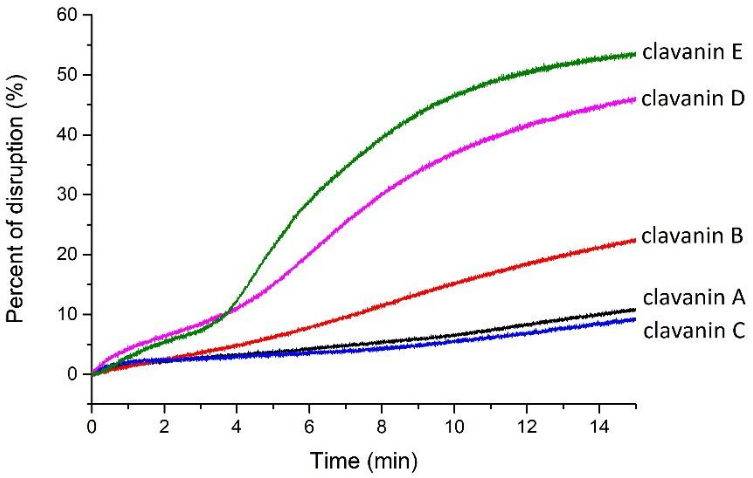


**Fig. S10.** Percentage of disrupted liposomes by: A) clavanin A; B) clavanin B; C) clavanin C; D) clavanin D; E) clavanin E. Diagram F) presents comparison of all tested clavanins. All samples were prepared in HEPES buffer (10 mM HEPES, 150 mM NaCl, pH = 7.4). Conditions: T = 298 K, [Cu(II)] = [Zn(II)] = [clavanin A] = [clavanin B] = [clavanin C] = [clavanin D] = [clavanin E] = 5 μM.

**Table S8.** Antibiotic resistance in indicated species of Gram-negative bacteria.

| Strain | No. | Biological source | Resistance mechanism | Aminoglycosides | Cephalosporins | Carbapenems | Penicillins with beta-lactamase inhibitors | Monobactams | Combination antibiotics |
|---|---|---|---|---|---|---|---|---|---|
| | | | | AN/TOB/GM | CIP/CAZ/CTX/CXM/FEP | IPM/MEM/DOR/ETP | AMC/SAM/TZP | ATM | SXT |
| *Escherichia coli* | HD41 | urine | ESβL | S/S/S | R/R/R/R/R | S/S/S/S | R/R/R | R | R |
| | HD84 | throat | ESβL | S/S/S | R/R/R/R/S | S/S/S/S | R/R/S | R | R |
| | Ped43 | foreskin | ESβL | S/S/R | R/R/R/R/R | S/S/S/S | R/R/S | R | R |
| | Ped47 | vagina | ESβL | I/R/R | R/R/R/R/R | S/S/S/S | R/R/R | R | R |

**Table S9.** Antibiotic resistance in indicated species of Gram-positive bacteria.

| Strain | No. | Biological source | Resistance mechanism | Aminoglycoside | Carbapenems | Penicillins | Oxazolidinones | Glycylcyclines | Glycopeptide antibiotics | Tetracyclines |
|---|---|---|---|---|---|---|---|---|---|---|
| | | | | GM/STR | IPM | AMP | LZD | TGC | VA | TEC |
| *Enterococcus faecalis* | HD30 | feces | HLAR, GRE | R/R | S | S | S | S | R | R |
| *Staphylococcus aureus MRSA* | HD27 | skin | MRSA, MLSβ | S/- | - | - | S | - | S | S |
| | HD33 | throat | MRSA, MLSβ | S/- | - | - | S | - | S | S |

R – resistant, I – intermediate, S – susceptible, (-) – not determined

**AN** - amikacin **TOB** - tobramycin, **GM** - gentamicin, **CIP** - ciprofloxacin, **CAZ** - ceftazidime, **CTX** - cefotaxim, **CXM** - cefuroxim, **FEP** - cefepime, **IPM** - imipenem, **MEM** - meropenem, **DOR** - doripenem, **ETP**- ertapenem, **AMC** - amoxicillin + clavulanic acid, **SAM** - ampicillin + sulbactam, **TZP** - piperacillin + tazobactam, **ATM** - aztreonam, **STR** - streptomycin, **SXT** - trimetoprim + sulfamethoxazole, **AMP** - ampicillin, **LZD** – linezolid, **TGC** – tigecycline, **VA** – vancomycin, **TEC** – tetracycline

**ESβL -** extended-spectrum beta-lactamases**, HLAR -** high-level aminoglycoside resistance**, GRE -** glycopeptide resistant Enterococci**, MRSA -** methicillin-resistant *Staphylococcus aureus*, **MLSβ** - macrolide-lincosamide-streptogramin B

# Abbreviations/Definitions

MIC - minimum inhibitory concentration/ minimum concentration, where microbial growth is inhibited

MBC - minimum bactericidal concentration/minimum concentration, where 99.9 % bacteria died

MFC - minimum fungicidal concentration/ minimum concentration, where 99.9 % fungi died

TTC - Triphenyl tetrazolium chloride

TSA – Tryptone Soy Agar

TSB - Tryptone Soy Broth

ATCC - American Type Culture Collections

EUCAST - European Committee on Antimicrobial Susceptibility Testing

ECACC - European Collection of Authenticated Cell Cultures

NR - Neutral Red

PBS - Phosphate Buffered Saline

MEMα – Minimum Essential Medium Eagle Alpha Modification

# References


1 G. Gran, *Acta Chem. Scand.*, 1950.

2 P. Gans and B. O'Sullivan, *Talanta*, 2000, 33–37.

3 P. Gans, A. Sabatini and A. Vacca, *Royal Society of Chemistry*, 1985.

4 L. Kacprzyk, V. Rydengård, M. Mörgelin, M. Davoudi, M. Pasupuleti, M. Malmsten and A. Schmidtchen, Antimicrobial activity of histidine-rich peptides is dependent on acidic conditions, *Biochimica et biophysica acta*, 2007, **1768**, 2667–2680.

5 J. R. Jimah, P. H. Schlesinger and N. H. Tolia, Liposome Disruption Assay to Examine Lytic Properties of Biomolecules, *Bio-protocol*, 2017, **7**. DOI: 10.21769/BioProtoc.2433.

6 Z. Mielke, Z. Latajka, A. Olbert-Majkut and R. Wieczorek, Matrix Infrared Spectra and ab Initio Calculations of the Nitrous Acid Complexes with Nitrogen Monoxide †, *J. Phys. Chem. A*, 2000, **104**, 3764–3769.

7 R. Wieczorek, Z. Latajka and J. Lundell, Quantum Chemical Study of the Bimolecular Complex of HONO, *J. Phys. Chem. A*, 1999, **103**, 6234–6239.

8 T. K. Olszewski, E. Wojaczyńska, R. Wieczorek and J. Bąkowicz, α-Hydroxyphosphonic acid derivatives of 2-azanorbornane: synthesis, DFT calculations, and crystal structure analysis, *Tetrahedron: Asymmetry*, 2015, **26**, 601–607.

9 P. Salvador, R. Wieczorek and J. J. Dannenberg, Direct calculation of trans-hydrogen-bond 13C-15N 3-bond J-couplings in entire polyalanine alpha-helices. A density functional theory study, *The journal of physical chemistry. B*, 2007, **111**, 2398–2403.

10 M. Rudowska, R. Wieczorek, A. Kluczyk, P. Stefanowicz and Z. Szewczuk, Gas-phase fragmentation of oligoproline peptide ions lacking easily mobilizable protons, *Journal of the American Society for Mass Spectrometry*, 2013, **24**, 846–856.

11 E. Gumienna-Kontecka, G. Berthon, I. O. Fritsky, R. Wieczorek, Z. Latajka and H. Kozlowski, 2-(Hydroxyimino)propanohydroxamic acid, a new effective ligand for aluminium, *J. Chem. Soc., Dalton Trans.*, 2000, 4201–4208.

12 R. Cammi, *Molecular Response Functions for the Polarizable Continuum Model. Physical basis and quantum mechanical formalism*, Springer International Publishing, Cham, 2013.

13 M. J. Frisch, G. W. Trucks, H. B. Schlegel, G. E. Scuseria, M. A. Robb, J. R. Cheeseman, G. Scalmani, V. Barone, G. A. Petersson, H. Nakatsuji, X. Li, M. Caricato, A. Marenich, J. Bloino, B. G. Janesko, R. Gomperts, B. Mennucci, H. P. Hratchian, J. V. Ortiz, A. F. Izmaylov, J. L. Sonnenberg, D. Williams-Young, F. Ding, F. Lipparini, F. Egidi, J. Goings, B. Peng, A. Petrone, T. Henderson, D. Ranasinghe, V. G.

Zakrzewski, J. Gao, N. Rega, G. Zheng, W. Liang, M. Hada, M. Ehara, K. Toyota, R. Fukuda, J. Hasegawa, M. Ishida, T. Nakajima, Y. Honda, O. Kitao, H. Nakai, T. Vreven, K. Throssell, J. A. Montgomery Jr., J. E. Peralta, F. Ogliaro, M. Bearpark, J. J. Heyd, E. Brothers, K. N. Kudin, V. N. Staroverov, T. Keith, R. Kobayashi, J. Normand, K. Raghavachari, A. Rendell, J. C. Burant, S. S. Iyengar, J. Tomasi, M. Cossi, J. M. Millam, M. Klene, C. Adamo, R. Cammi, J. W. Ochterski, R. L. Martin, K. Morokuma, O. Farkas, J. B. Foresman and D. J. Fox, *Gaussian 09*, Gaussian, Inc., Wallingford CT, 2016.

14 J.-D. Chai and M. Head-Gordon, Long-range corrected hybrid density functionals with damped atom-atom dispersion corrections, *Physical chemistry chemical physics : PCCP*, 2008, **10**, 6615–6620.

15 M. P. Weinstein, *Methods for dilution antimicrobial susceptibility tests for bacteria that grow aerobically*, National Committee for Clinical Laboratory Standards, Villanova, Pennsylvania, 11th edn., 2018.

16 International Organization for Standarization, *Susceptibility testing of infectious agents and evaluation of performance of antimicrobial susceptibility test devices – Part 1: Broth micro-dilution reference method for testing the in vitro activity of antimicrobial agents against rapidly growing aerobic bacteria involved in infectious diseases*, 2019.

17 International Organization for Standarization, *Clinical laboratory testing and in vitro diagnostic test systems – Reference method for testing the in vitro activity of antimicrobial agents against yeast fungi involved in infectious diseases*, 2012.

18 J. Gabrielson, M. Hart, A. Jarelöv, I. Kühn, D. McKenzie and R. Möllby, Evaluation of redox indicators and the use of digital scanners and spectrophotometer for quantification of microbial growth in microplates, *Journal of microbiological methods*, 2002, **50**, 63–73.

19 F. L. Francisco, A. M. Saviano, T. d. J. A. Pinto and F. R. Lourenço, Development, optimization and validation of a rapid colorimetric microplate bioassay for neomycin sulfate in pharmaceutical drug products, *Journal of microbiological methods*, 2014, **103**, 104–111.

20 P. Sabaeifard, A. Abdi-Ali, M. R. Soudi and R. Dinarvand, Optimization of tetrazolium salt assay for Pseudomonas aeruginosa biofilm using microtiter plate method, *Journal of microbiological methods*, 2014, **105**, 134–140.

21 G. Repetto, A. del Peso and J. L. Zurita, Neutral red uptake assay for the estimation of cell viability/cytotoxicity, *Nature protocols*, 2008, **3**, 1125–1131.

22 The European Committee on Antimicrobial Susceptibility Testing, *Breakpoint tables for interpretation of MICs and zone diameters,* 11th edn., 2021, https://www.eucast.org/clinical_breakpoints/.

23 S. A. Juliano, S. Pierce, J. A. deMayo, M. J. Balunas and A. M. Angeles-Boza, Exploration of the Innate Immune System of Styela clava: Zn2+ Binding Enhances the Antimicrobial Activity of the Tunicate Peptide Clavanin A, *Biochemistry*, 2017, **56**, 1403–1414.
